# Runs of homozygosity in Sable Island feral horses reveal the genomic consequences of inbreeding and divergence from domestic breeds

**DOI:** 10.1186/s12864-022-08729-9

**Published:** 2022-07-12

**Authors:** Julie Colpitts, Philip Dunstan McLoughlin, Jocelyn Poissant

**Affiliations:** 1 Department of Biology, University of Saskatchewan, Saskatchewan, Canada; 2 Faculty of Veterinary Medicine, University of Calgary, Calgary, Alberta Canada

**Keywords:** Signatures of selection, ROH islands, Gene ontology enrichment, Genetic reservoir, Conservation genomics

## Abstract

**Background:**

Understanding inbreeding and its impact on fitness and evolutionary potential is fundamental to species conservation and agriculture. Long stretches of homozygous genotypes, known as runs of homozygosity (ROH), result from inbreeding and their number and length can provide useful population-level information on inbreeding characteristics and locations of signatures of selection. However, the utility of ROH for conservation is limited for natural populations where baseline data and genomic tools are lacking. Comparing ROH metrics in recently feral vs. domestic populations of well understood species like the horse could provide information on the genetic health of those populations and offer insight into how such metrics compare between managed and unmanaged populations. Here we characterized ROH, inbreeding coefficients, and ROH islands in a feral horse population from Sable Island, Canada, using ~41 000 SNPs and contrasted results with those from 33 domestic breeds to assess the impacts of isolation on ROH abundance, length, distribution, and ROH islands.

**Results:**

ROH number, length, and ROH-based inbreeding coefficients (F_ROH_) in Sable Island horses were generally greater than in domestic breeds. Short runs, which typically coalesce many generations prior, were more abundant than long runs in all populations, but run length distributions indicated more recent population bottlenecks in Sable Island horses. Nine ROH islands were detected in Sable Island horses, exhibiting very little overlap with those found in domestic breeds. Gene ontology (GO) enrichment analysis for Sable Island ROH islands revealed enrichment for genes associated with 3 clusters of biological pathways largely associated with metabolism and immune function.

**Conclusions:**

This study indicates that Sable Island horses tend to be more inbred than their domestic counterparts and that most of this inbreeding is due to historical bottlenecks and founder effects rather than recent mating between close relatives. Unique ROH islands in the Sable Island population suggest adaptation to local selective pressures and/or strong genetic drift and highlight the value of this population as a reservoir of equine genetic variation. This research illustrates how ROH analyses can be applied to gain insights into the population history, genetic health, and divergence of wild or feral populations of conservation concern.

**Supplementary Information:**

The online version contains supplementary material available at 10.1186/s12864-022-08729-9.

## Background

It has long been recognized that understanding inbreeding is crucial to the goals of conservation, wildlife management and livestock breeding programs. Elevated levels of inbreeding in vulnerable populations can compromise their long-term viability and undermine conservation efforts if not actively mitigated [1], while strong artificial selection for specific traits in livestock species typically exacerbates inbreeding as a side effect and can be counter-productive if fitness is negatively impacted [2]. However, decreased genetic diversity is not guaranteed to have negative fitness consequences (e.g. strong directional selection will decrease genetic diversity across the genome – in particular in regions directly under selection – while increasing fitness), so characterizing what changes in diversity look like at the genomic level is crucial for assessing genetic health and viability of both wildlife and livestock populations.

One approach for assessing inbreeding in individuals and populations is characterizing runs of homozygosity (ROH). ROH are continuous lengths of homozygous genotypes which result from inbreeding when identical haplotypes are inherited from both parents (i.e. identical by descent [3]). It is expected that the mating of closely related individuals will cause many long ROH in resulting offspring due to the limited number of crossovers occurring during meiosis, but in the absence of continuous inbreeding haplotypes will be broken down over time, leading to shorter ROH and making it possible to surmise the relative coalescence time of haplotypes (sometimes referred to as the “age” of inbreeding) based on the length of detectable runs [3]. Additionally, ROH can result from natural and artificial selection as the frequency of haplotypes associated with traits being selected for increases in a population. This leads to ROH islands, or areas of the genome where ROH are more abundant than would be expected in the absence of selection [4]. ROH therefore not only provide information on the inbreeding level and history of individuals and populations, but also on genomic regions and genes impacted by selection.

Assessments of ROH have become widespread in agriculturally important species such as sheep, cattle, goats and pigs (e.g. [5–8]). For example, Martikainen et al. [9] were able to identify ROH associated with decreased fertility and milk production in female Ayrshire cattle, while Purfield et al. [5] identified signatures of selection for pigmentation, body size and muscle formation in ROH of a variety of meat sheep breeds. Mastrangelo et al. [6] characterized autozygosity in 21 Italian sheep breeds, and work on population histories using ROH has been done in cattle since at least 2012 [10]. ROH studies in horses are so far less common and range from determining breed history in one to three breeds ([11, 12] respectively), assessing genetic architecture of complex traits in the Lipizzan horse [13], and revealing signatures of selection in 10 individuals from various breed origins [14]. Most recently, a repository of ROH islands became available for thirty-five domestic horse breeds [15], but knowledge of how this compares to their feral counterparts is lacking.

In contrast to livestock species, relatively little has been done on ROH in wildlife despite their potential to inform conservation [16]. This is likely because calculation of ROH requires a reasonable genome assembly and a large number of genetic markers, which are still relatively difficult to generate for wildlife. While these studies begin to emerge (see [17] for one such example exploring killer whale demography, [18] for a study investigating ROH in an inbred wolf population and [19] for a study of the genetic landscape in red deer), characterizing runs of homozygosity is currently more feasible in wild or feral populations of agriculturally important species for which genome assemblies and high-throughput genotyping arrays are readily available. This has been explored to some extent with wild boars, feral pigs, and Soay sheep, for example [20–22].

**Fig. 1:**
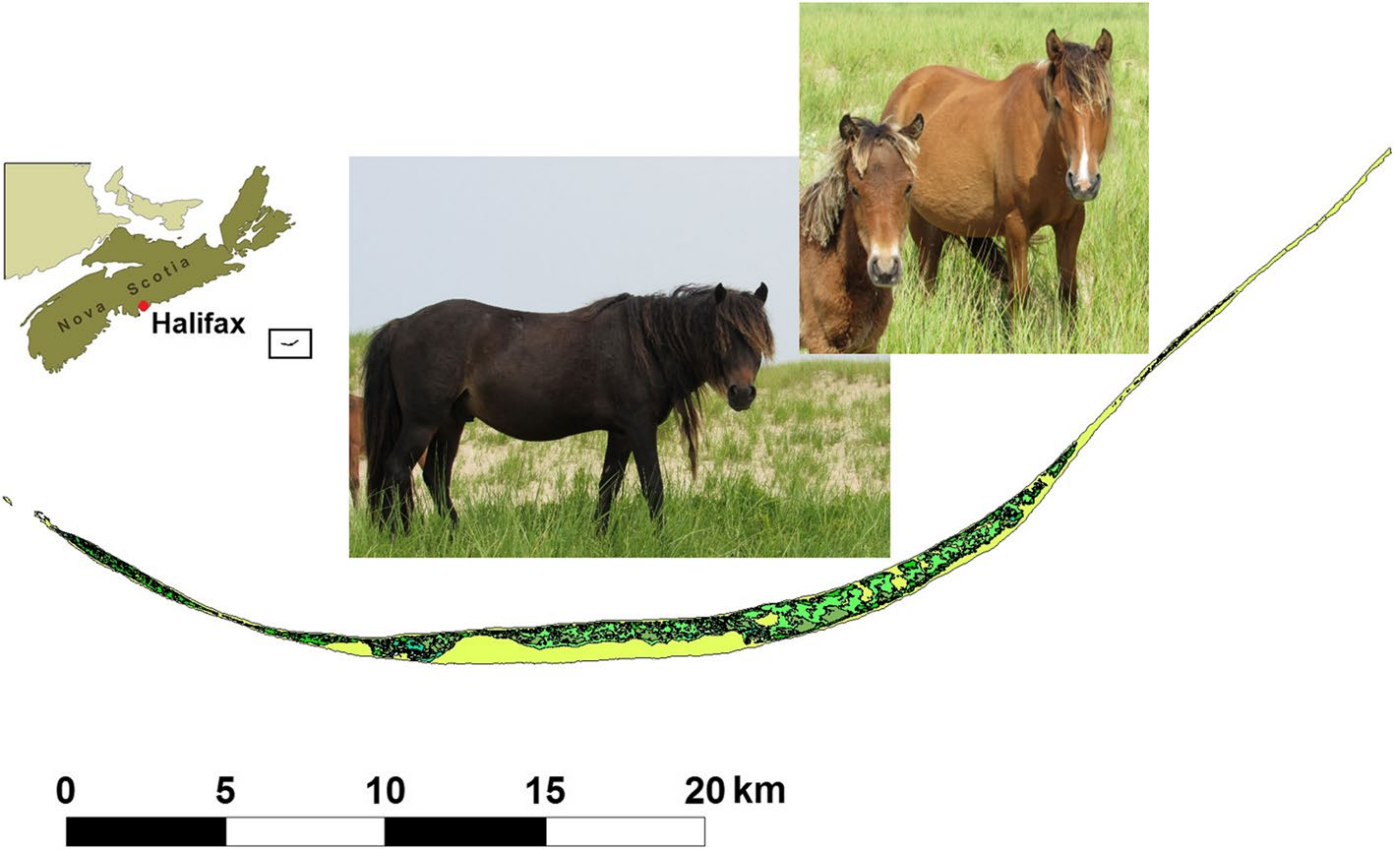
Map of Sable Island. Depiction of the island along with its location relative to Halifax, Nova Scotia and representative photos of Sable Island horses (left: stallion, right: adult mare with juvenile)

Many feral horse populations exist throughout the world, with varying degrees of isolation and management practices [23]. One such population exists on Sable Island, Nova Scotia, Canada (Fig. 1). This population was established through numerous introductions dating back to the second half of the 18^th^ century, possibly sourced from horses confiscated from French settlers during the Acadian expulsion of 1755 [24]. Genetic studies conducted thus far indicate that the population is most closely related to horses of Nordic origins [25, 26]. The small (≈250 – 550) unmanaged population has been isolated from any known admixture since 1935 [27], and protected from all human interference since 1960 [24, 25].

Previous research on Sable Island horses has shown that genetic diversity in the population is low [25], and effective population size (N_e_) has been estimated at approximately 48 individuals [28]. However, little is known about the history and genomic consequences of inbreeding in the population, or to what extent genetic drift plays a role in defining genomic characteristics. Further, this population is subject to natural selection in the absence of predators and survives in unpredictable and harsh conditions, but little is known about how this manifests at the genetic level and to what extent these horses may serve as a reservoir of useful equine genetic variation. In this study, we characterized ROH abundance, length and location in the Sable Island horse population using commercial SNP arrays and contrasted results with those from publicly available genotypes from a large number of domestic breeds using a common set of loci. Our goals were to determine if historical and recent patterns of inbreeding differed between Sable Island horses and domestic breeds, if ROH islands found in Sable Island horses were unique to this population, and if genes located within ROH islands could provide insights into the population’s adaptation to its unique environment.

## Results

### ROH abundance

**Fig. 2:**
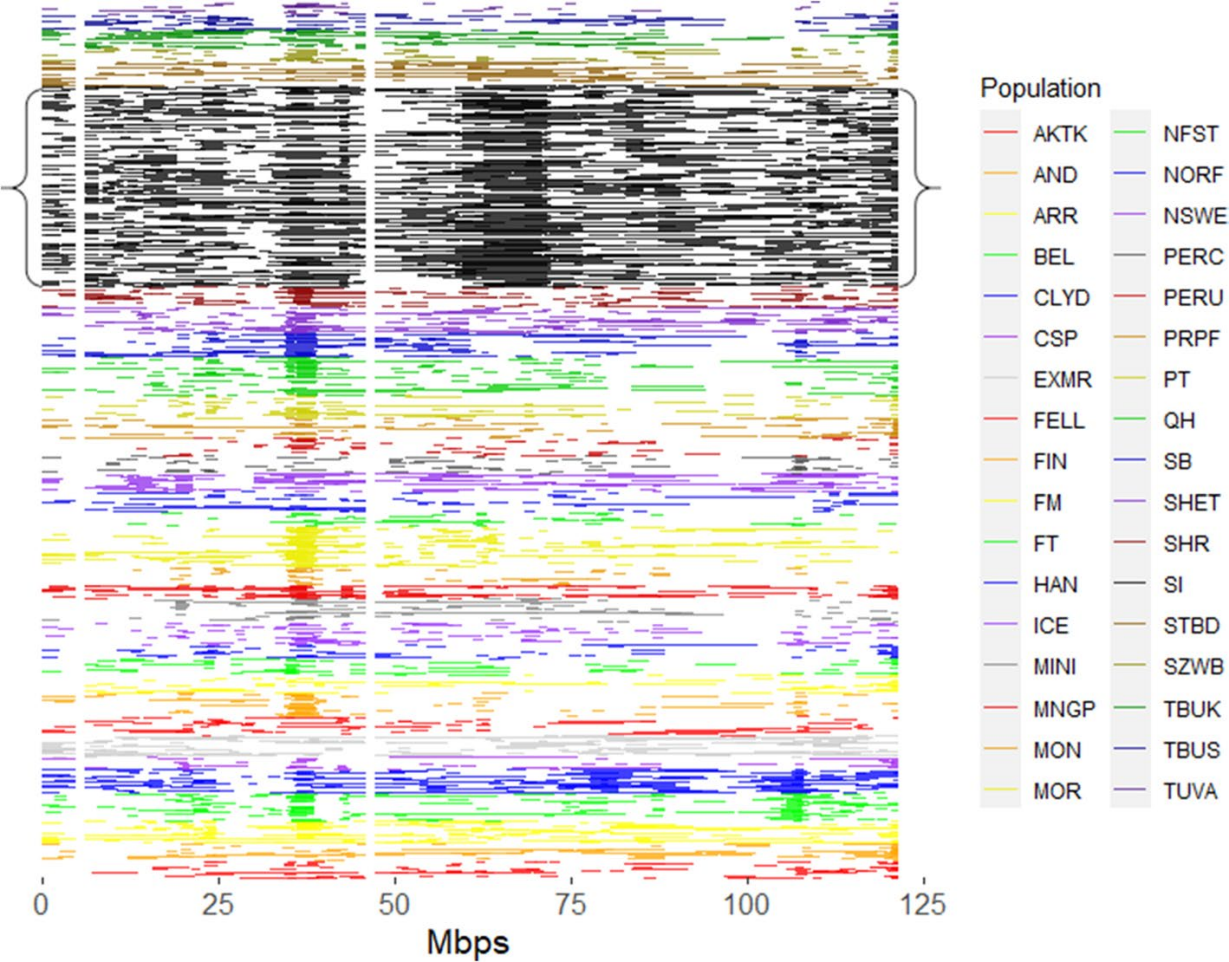
ROH plotted along chromosome 3. All ROH detected on chromosome 3 for 33 breeds of domestic horses and Sable Island feral horses. Each individual is represented along the y axis and each horizontal line indicates the length and location of runs of homozygosity for each individual. Each population is indicated by a different colour as abbreviated in the legend and Sable Island horses are indicated in black and encompassed by curly brackets. Horse populations in ascending order are as follows: Akhal Teke, Andalusian, Arabian, Belgian, Clydesdale, Caspian, Exmoor, Fell Pony, Finnhorse, Franches-Montagnes, French Trotter, Hanovarian, Icelandic, Miniature, Mangalara Paulista, Mongolian, Morgan, New Forest Pony, Norwegian Fjord, North Swedish Horse, Percheron, Peruvian Paso, Puerto Rican Paso Fina, Paint, Quarter Horse, Saddlebred, Shetland, Shire, Sable Island feral horses, Standardbred, Swiss Warmblood, European Thoroughbred, American Thoroughbred and Tuva

Runs of homozygosity were found in all individuals of all groups of horses, and occurred throughout the genome. An exemplary visual representation of the number, length and distribution of ROH on chromosome 3 can be seen in Fig. 2. The average number of runs in Sable Island horses was 139 and ranged from 39 to 131 in domestic breeds (Table 1). The number of ROH per individual ranged from 109 to 212 in Sable Island horses, and 13 to 228 in domestic breeds (Table 1).

**Table 1:** Mean number and length of ROH and genome-wide ROH-based inbreeding coefficients

Population (n)	Mean N ROH	Mean ROH length (Mb)	Mean F_ROH_
	(min, max)	(min, max)	(min, max)
Sable Island (212)	139 (109, 212)	4.72 (2.50, 7.23)	0.29 (0.16, 0.42)
Clydesdale (24)	131 (99, 155)	4.94 (3.16, 6.02)	0.29 (0.14, 0.35)
Mangalara Paulista (14)	119 (89, 167)	5.00 (2.99, 6.39)	0.26 (0.19, 0.34)
Exmoor (24)	121 (65, 205)	4.72 (2.95, 7.49)	0.25 (0.08, 0.52)
Thoroughbred (Europe) (19)	121 (104, 139)	4.10 (3.66, 4.59)	0.22 (0.19, 0.25)
Thoroughbred (US) (17)	119 (97, 137)	4.11 (3.54, 5.14)	0.21 (0.17, 0.26)
Shetland (27)	118 (89, 228)	3.65 (2.76, 5.92)	0.19 (0.12, 0.32)
Standardbred (25)	87 (69, 102)	4.84 (3.45, 5.87)	0.19 (0.12, 0.24)
Arabian (24)	105 (84, 127)	3.67 (2.53, 7.48)	0.17 (0.09, 0.38)
Andalusian (18)	74 (52, 99)	5.02 (2.97, 9.68)	0.17 (0.08, 0.35)
Shire (22)	117 (24, 173)	3.35 (1.99, 4.37)	0.17 (0.02, 0.26)
French Trotter (17)	83 (71, 104)	4.30 (3.14, 5.42)	0.16 (0.10, 0.21)
North Swedish Horse (19)	89 (70, 132)	3.75 (2.85, 5.89)	0.15 (0.09, 0.23)
Norwegian Fjord (21)	78 (57, 96)	4.36 (3.17, 6.07)	0.15 (0.09, 0.21)
Saddlebred (25)	73 (55, 90)	4.41 (3.45, 5.80)	0.14 (0.09, 0.20)
Fell Pony (21)	79 (63, 109)	4.03 (2.81, 5.81)	0.14 (0.09, 0.19)
Franches-Montagnes (19)	73 (56, 91)	3.93 (3.35, 5.89)	0.13 (0.08, 0.23)
Belgian (30)	84 (58, 106)	3.54 (2.57, 4.68)	0.13 (0.08, 0.17)
Morgan (43)	67 (41, 113)	4.31 (2.51, 10.84)	0.13 (0.06, 0.33)
Puerto Rican Paso Fino (20)	79 (44, 103)	3.72 (1.97, 7.00)	0.13 (0.04, 0.32)
Akhal Teke (19)	80 (62, 97)	3.36 (2.48, 4.29)	0.12 (0.08, 0.16)
Swiss Warmblood (14)	78 (62, 101)	3.23 (2.73, 4.29)	0.11 (0.08, 0.19)
Hanoverian (15)	75 (48, 95)	3.21 (2.38, 3.78)	0.11 (0.05, 0.15)
Icelandic (25)	85 (70, 99)	2.65 (2.15, 5.71)	0.10 (0.07, 0.25)
Quarter Horse (40)	67 (51, 103)	3.42 (2.24, 6.01)	0.10 (0.06, 0.18)
Miniature (25)	71 (55, 91)	3.08 (1.98, 10.12)	0.10 (0.05, 0.34)
Percheron (20)	81 (49, 105)	2.74 (2.12, 3.74)	0.10 (0.05, 0.14)
Paint (25)	67 (46, 100)	3.08 (2.42, 4.57)	0.09 (0.06, 0.16)
Peruvian Paso (21)	62 (41, 77)	3.29 (2.11, 5.25)	0.09 (0.05, 0.16)
Finnhorse (27)	46 (29, 60)	3.21 (1.89, 4.72)	0.07 (0.03, 0.12)
Caspian (14)	48 (13, 95)	2.43 (1.81, 3.81)	0.06 (0.01, 0.11)
New Forest Pony (15)	44 (35, 56)	2.80 (2.18, 4.47)	0.05 (0.04, 0.10)
Tuva (15)	39 (24, 51)	2.83 (1.72, 6.28)	0.05 (0.02, 0.14)

Values shown for individuals from 33 domestic horse breeds and Sable Island feral horses. Sample sizes and data ranges are shown in parentheses. Breeds are ordered by F_ROH_ values

**Fig. 3:**
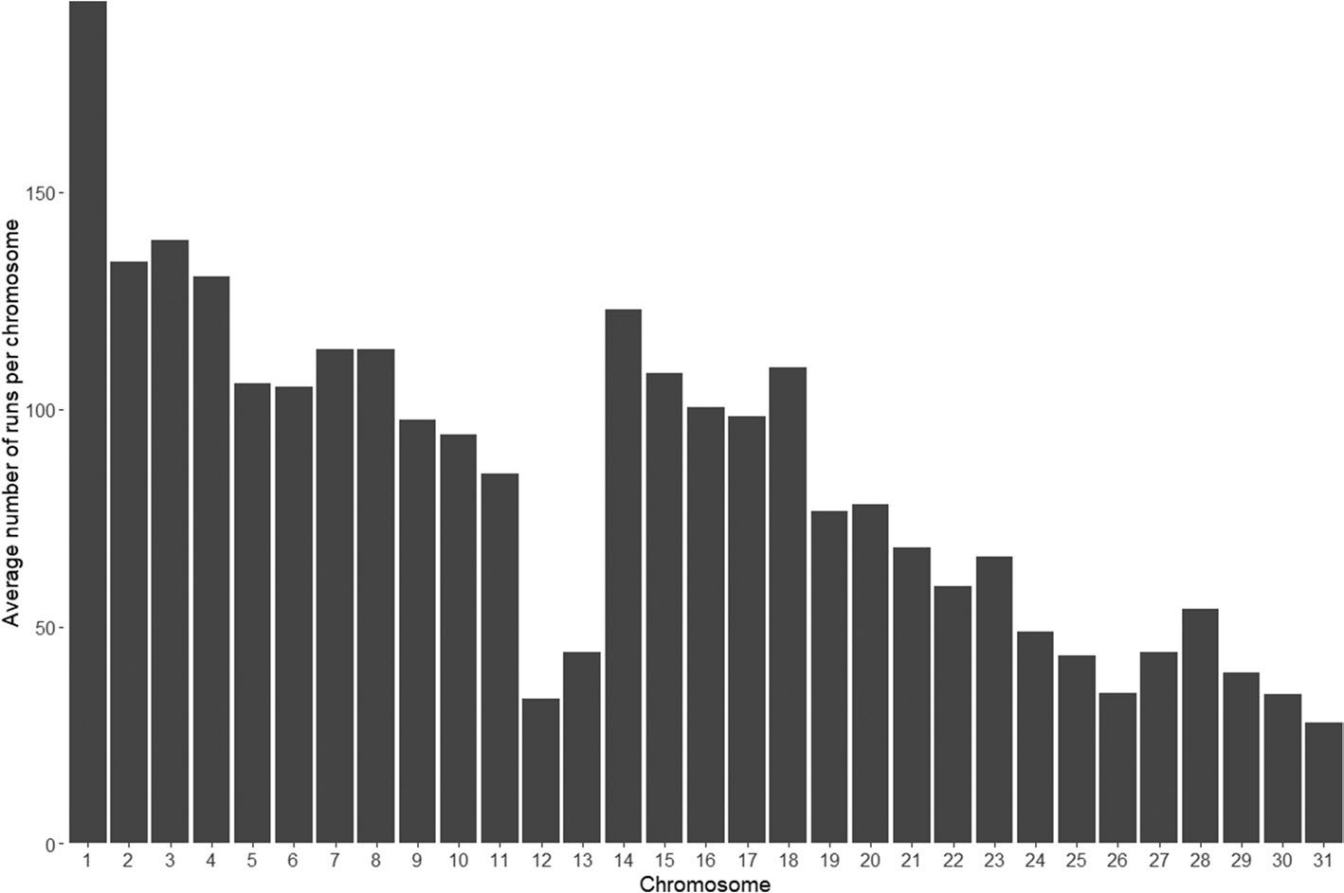
Average number of ROH across all horse populations studied for each chromosome

In Sable Island horses, the average number of runs per chromosome ranged from 1.69 (ECA30) to 9.82 (ECA1), while in domestic breeds the average ranged from 0.80 (ECA31) to 6.24 (ECA1). The number of runs per chromosome generally increased with chromosome length (R^2^=0.68), but notably, chromosomes 12 and 13 had substantially fewer ROH than would be expected from this overall trend (R^2^=0.82 when those 2 chromosomes are excluded; see Fig. 3 for overall trend).

### ROH length

The length of ROH across all studied horses ranged from 0.57 to 84.01 Mb (both in domestic breeds) and averaged 3.7 Mb. The overall average length of runs in Sable Island horses was 4.72 Mb while it ranged from 1.99 to 5.02 Mb in domestic breeds (Table 1). The average ROH length per individual ranged from 2.5 to 7.23 Mb in Sable Island horses, and from 1.72 to 10.84 Mb in domestic breeds.

**Fig. 4:**
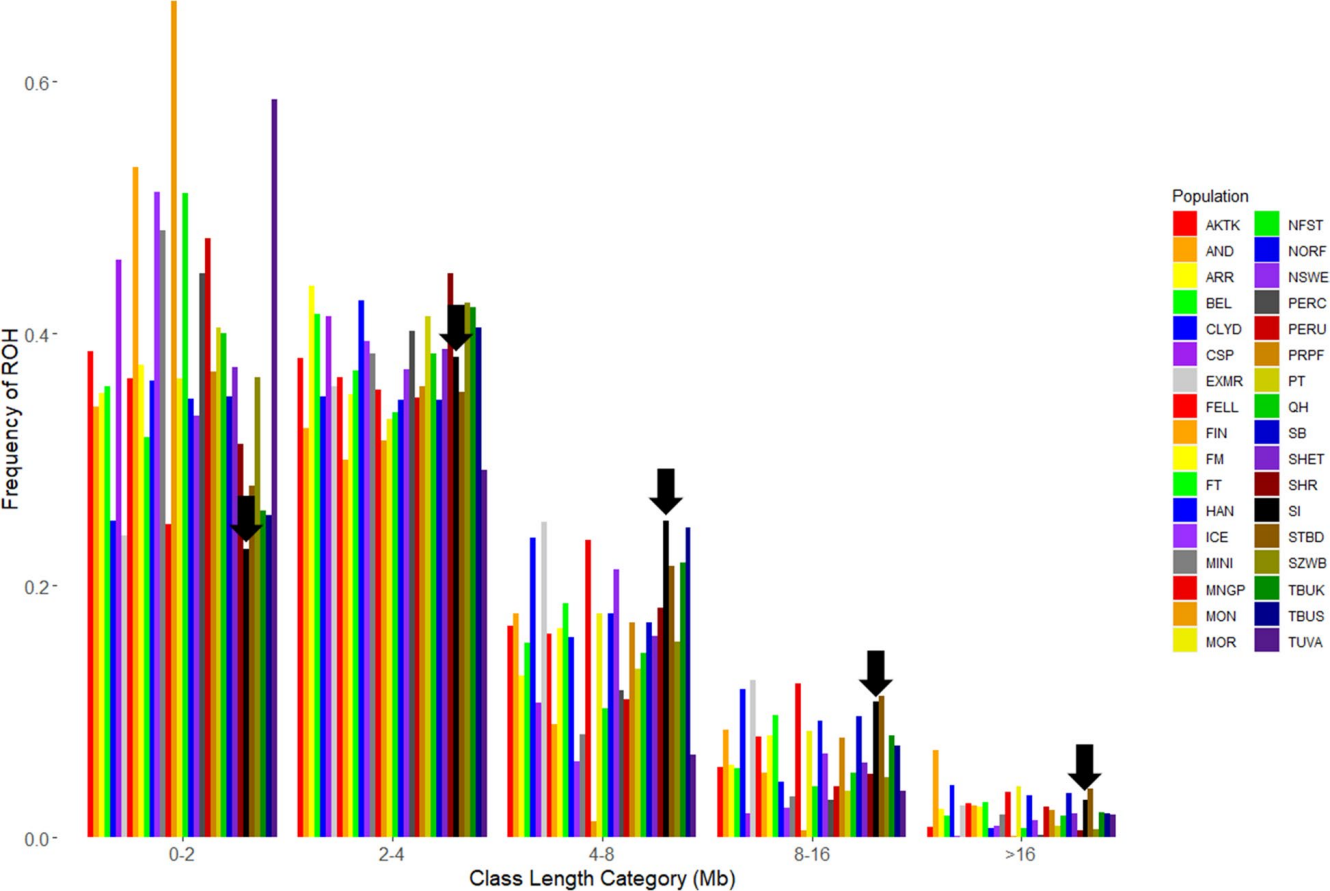
Distribution of ROH in percentage of runs within each length class. ROH according to run length category in Mb for individuals from 33 domestic horse breeds and Sable Island feral horses. Each population is represented by a different colour as abbreviated in the legend and Sable Island results are indicated with black arrows. Horse populations in order of appearance are as follows: Akhal Teke, Andalusian, Arabian, Belgian, Clydesdale, Caspian, Exmoor, Fell Pony, Finnhorse, Franches-Montagnes, French Trotter, Hanovarian, Icelandic, Miniature, Mangalara Paulista, Mongolian, Morgan, New Forest Pony, Norwegian Fjord, North Swedish Horse, Percheron, Peruvian Paso, Puerto Rican Paso Fina, Paint, Quarter Horse, Saddlebred, Shetland, Shire, Sable Island feral horses, Standardbred, Swiss Warmblood, European Thoroughbred, American Thoroughbred and Tuva

Although the relative proportions of run lengths varied across populations, all distributions were skewed towards shorter runs (Fig. 4). Notably, Sable Island horses had the smallest proportion of runs 0-2 Mb in length and the highest proportion of runs 4-8 Mb long. In Sable Island horses, 23% of ROH appeared in the 0-2 Mb length category while the overall average proportion of ROH this length was 38% (Fig. 4). Conversely, 25% of all runs in Sable Island horses fell into the 4-8 Mb length category while the overall average proportion of runs in this length class was 15% (Fig. 4). At the individual level many of the domestic breeds had at least one individual which possessed longer ROH than the average Sable Island horse (see Table 1 for data ranges). Run length and therefore coalescence time appears to be more variable in many domestic breeds than in Sable Island feral horses.

Unlike ROH abundance, average run length did not vary with any discernible pattern according to chromosome size (R^2^=0.09). In Sable Island horses, average per-chromosome run length ranged from 3.55 Mb on ECA12 to 6.03 Mb on ECA23 and 3.16 Mb on ECA31 to 4.34 Mb on ECA26 in domestic breeds.

### Inbreeding

Average ROH-based inbreeding coefficients (F_ROH_) derived from the amount of the genome present within all lengths of ROH vs total genome length ranged from 0.03 in Mongolian horses to 0.29 in Sable Island horses and Clydesdales (Table 1). Chromosome-specific F_ROH_ was highly variable, but Sable Island had among the highest F_ROH_ values for all chromosomes (Additional file 1). In particular, Sable Island had the highest mean F_ROH_ for chromosomes 1, 3, 14, 18, 20, 23 and 31 (Additional file 1).

As is typical, shorter runs were more abundant than long ones for each horse population studied and contributed more to inbreeding metrics. In all cases, when F_ROH_ was calculated with increasing run length thresholds, F_ROH_ and the number of individuals for which it could be calculated decreased (Table 2). As long as runs of 4 Mb or shorter were included, Sable Island horses had the highest average F_ROH_ of all breeds (0.29 and 0.26 for the shortest runs length classes, respectively; Table 2). Sable Island horses were again among the most inbred in intermediate run length classes with F_ROH_ of 0.20 for runs > 4 Mb and 0.11 for runs >8 Mb (Table 2). When only very long ROH (>16 Mb) were considered, average F_ROH_ was 0.04 for Sable Island (range 0.01 to 0.22) and values were very small in domestic breeds as well (Table 2).

**Table 2:** Mean ROH-based inbreeding coefficient (F_ROH_) by run length class

Population (n)	F_ROH_ > 0 Mb	F_ROH_ > 2 Mb	F_ROH_ > 4 Mb	F_ROH_ > 8 Mb	F_ROH_ > 16 Mb
	Mean (min, max)	Mean (min, max)	Mean (min, max)	Mean (min, max)	Mean (min, max)
Sable Island (212)	0.29 (0.16, 0.42)	0.26 (0.13, 0.41)	0.20 (0.04, 0.35)	0.11 (0.00, 0.28)	0.04 (0.01, 0.22)
Clydesdale (24)	0.29 (0.14, 0.35)	0.26 (0.11, 0.33)	0.21 (0.06, 0.27)	0.13 (0.02, 0.21)	0.05 (0.01, 0.10)
Mangalara Paulista (14)	0.26 (0.19, 0.34)	0.24 (0.17, 0.31)	0.18 (0.08, 0.26)	0.11 (0.01, 0.20)	0.05 (0.01, 0.10)
Exmoor (24)	0.25 (0.08, 0.52)	0.23 (0.06, 0.50)	0.18 (0.04, 0.43)	0.10 (0.02, 0.27)	0.04 (0.01, 0.13)
Thoroughbred (Europe) (19)	0.22 (0.19, 0.25)	0.20 (0.17, 0.23)	0.13 (0.11, 0.16)	0.07 (0.04, 0.11)	0.02 (0.01, 0.05)
Thoroughbred (US) (17)	0.21 (0.17, 0.26)	0.19 (0.15, 0.23)	0.13 (0.09, 0.17)	0.06 (0.04, 0.10)	0.02 (0.01, 0.07)
Shetland (27)	0.19 (0.12, 0.32)	0.16 (0.09, 0.30)	0.10 (0.04, 0.24)	0.06 (0.01, 0.18)	0.04 (0.01, 0.14)
Standardbred (25)	0.19 (0.12, 0.24)	0.17 (0.10, 0.23)	0.13 (0.07, 0.19)	0.08 (0.03, 0.13)	0.04 (0.01, 0.07)
Arabian (24)	0.17 (0.09, 0.38)	0.14 (0.07, 0.36)	0.09 (0.02, 0.32)	0.06 (0.01, 0.28)	0.03 (0.01, 0.18)
Andalusian (18)	0.17 (0.08, 0.35)	0.15 (0.06, 0.34)	0.12 (0.04, 0.31)	0.09 (0.01, 0.27)	0.06 (0.01, 0.23)
Shire (22)	0.17 (0.02, 0.26)	0.15 (0.01, 0.22)	0.08 (0.00, 0.16)	0.04 (0.00, 0.08)	0.02 (0.01, 0.02)
French Trotter (17)	0.16 (0.10, 0.21)	0.14 (0.08, 0.20)	0.10 (0.04, 0.16)	0.06 (0.02, 0.10)	0.03 (0.01, 0.06)
North Swedish Horse (19)	0.15 (0.09, 0.23)	0.13 (0.07, 0.21)	0.09 (0.03, 0.17)	0.04 (0.00, 0.12)	0.02 (0.01, 0.08)
Norwegian Fjord (21)	0.15 (0.09, 0.21)	0.13 (0.08, 0.20)	0.10 (0.04, 0.15)	0.06 (0.02, 0.11)	0.03 (0.01, 0.07)
Saddlebred (25)	0.14 (0.09, 0.20)	0.12 (0.08, 0.18)	0.09 (0.05, 0.15)	0.06 (0.02, 0.11)	0.03 (0.01, 0.07)
Fell Pony (21)	0.14 (0.09, 0.19)	0.12 (0.08, 0.17)	0.08 (0.04, 0.14)	0.05 (0.00, 0.12)	0.03 (0.01, 0.07)
Franches-Montagnes (19)	0.13 (0.08, 0.23)	0.11 (0.07, 0.22)	0.08 (0.04, 0.18)	0.05 (0.01, 0.14)	0.03 (0.01, 0.08)
Belgian (30)	0.13 (0.08, 0.17)	0.11 (0.05, 0.15)	0.07 (0.02, 0.11)	0.04 (0.00, 0.08)	0.02 (0.01, 0.05)
Morgan (43)	0.13 (0.06, 0.33)	0.11 (0.04, 0.32)	0.09 (0.01, 0.30)	0.06 (0.00, 0.28)	0.04 (0.01, 0.21)
Puerto Rican Paso Fino (20)	0.13 (0.04, 0.32)	0.11 (0.02, 0.30)	0.08 (0.00, 0.27)	0.05 (0.00, 0.22)	0.03 (0.01, 0.11)
Akhal Teke (19)	0.12 (0.08, 0.16)	0.10 (0.05, 0.14)	0.06 (0.02, 0.10)	0.03 (0.00, 0.06)	0.01 (0.01, 0.03)
Swiss Warmblood (14)	0.11 (0.08, 0.19)	0.09 (0.06, 0.17)	0.05 (0.03, 0.12)	0.02 (0.00, 0.07)	0.01 (0.01, 0.03)
Hanoverian (15)	0.11 (0.05, 0.15)	0.09 (0.04, 0.13)	0.05 (0.01, 0.08)	0.02 (0.00, 0.05)	0.01 (0.01, 0.02)
Icelandic (25)	0.10 (0.07, 0.25)	0.07 (0.04, 0.22)	0.03 (0.00, 0.17)	0.02 (0.00, 0.15)	0.03 (0.01, 0.13)
Quarter Horse (40)	0.10 (0.06, 0.18)	0.08 (0.03, 0.16)	0.05 (0.01, 0.14)	0.03 (0.00, 0.12)	0.02 (0.01, 0.08)
Miniature (25)	0.10 (0.05, 0.34)	0.07 (0.03, 0.33)	0.04 (0.00, 0.30)	0.03 (0.00, 0.28)	0.05 (0.01, 0.25)
Percheron (20)	0.10 (0.05, 0.14)	0.07 (0.03, 0.12)	0.03 (0.00, 0.08)	0.02 (0.00, 0.06)	0.01 (0.01, 0.02)
Paint (25)	0.09 (0.06, 0.16)	0.07 (0.04, 0.14)	0.04 (0.02, 0.10)	0.02 (0.00, 0.07)	0.02 (0.01, 0.04)
Peruvian Paso (21)	0.09 (0.05, 0.16)	0.07 (0.02, 0.14)	0.05 (0.01, 0.12)	0.04 (0.00, 0.09)	0.03 (0.01, 0.07)
Finnhorse (27)	0.07 (0.03, 0.12)	0.05 (0.01, 0.11)	0.03 (0.00, 0.08)	0.02 (0.01, 0.06)	0.01 (0.01, 0.04)
Caspian (14)	0.06 (0.01, 0.11)	0.04 (0.00, 0.10)	0.03 (0.00, 0.07)	0.01 (0.00, 0.03)	0.01 (0.01, 0.01)
New Forest Pony (15)	0.05 (0.04, 0.10)	0.04 (0.02, 0.09)	0.02 (0.01, 0.06)	0.01 (0.00, 0.05)	0.01 (0.01, 0.02)
Tuva (15)	0.05 (0.02, 0.14)	0.04 (0.01, 0.12)	0.03 (0.00, 0.11)	0.04 (0.00, 0.10)	0.03 (0.01, 0.07)
Mongolian (19)	0.03 (0.02, 0.05)	0.01 (0.01, 0.04)	0.01 (0.00, 0.03)	0.01 (0.01, 0.02)	0.01 (0.01, 0.01)

Values shown for individuals from 33 domestic horse breeds and Sable Island feral horses. Sample sizes and data ranges are shown in parentheses. Breeds are ordered by overall F_ROH_ value

**Fig. 5:**
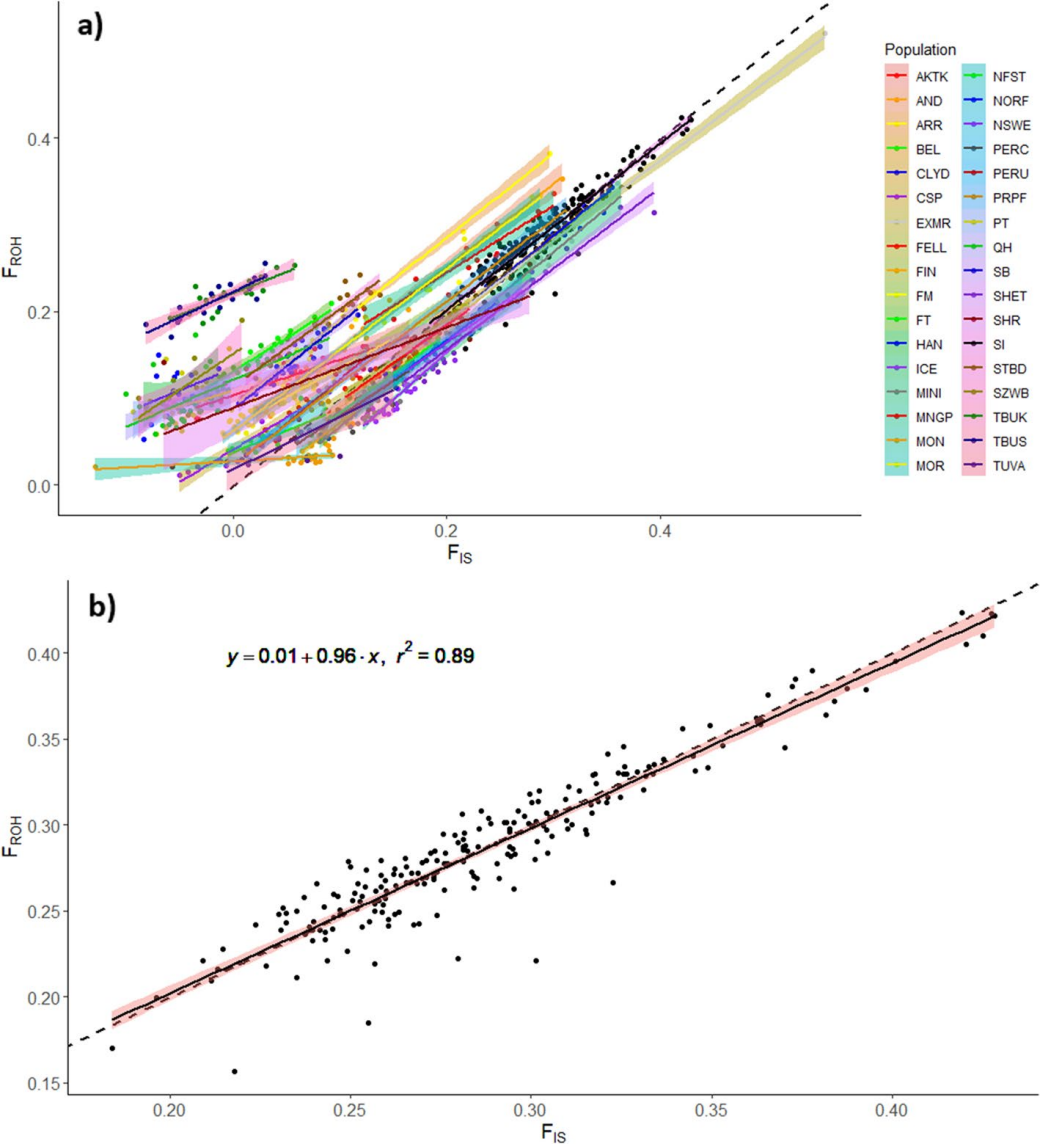
Genome-wide F_ROH_ vs F_IS_. Values are plotted for all individuals of (**a**) 33 domestic horse breeds and Sable Island feral horses and (**b**) Sable Island horses only. Corresponding linear trendlines with 95% confidence intervals are shown for all horse populations, and the trendline equation is presented for Sable Island horses. F_ROH_ is the summed length of all ROH divided by total genome length whereas F_IS_ is a measure of expected vs observed homozygosity and provides a measure of non-random mating in the most recent generation (F_IS_ = 0 indicates random mating, F_IS_ > 0 indicates consanguinity and F_IS_ < 0 indicates inbreeding avoidance). The dashed line on both plots indicates where F_ROH_ = F_IS_, along which all excess homozygosity is accounted for by ROH. Points along the Y axis would indicate ROH caused primarily by small N_e_ while those along the X axis would indicate elevated admixture

To validate the use of F_ROH_ as a measure of consanguinity and provide insight into population structure, an additional inbreeding coefficient (F_IS_) was calculated for all individuals. F_ROH_ and F_IS_ were correlated to varying degrees in each breed studied (Fig. 5a) with a large number of domestic breeds having a slightly higher than expected F_ROH_ to F_IS_ ratio. Sable Island horses showed strong correlation between F_ROH_ and F_IS_ (r^2^ = 0.89; Fig. 5b), and most individuals fell along the unity line where F_ROH_ = F_IS_.

### Signatures of selection and GO analysis

**Fig. 6:**
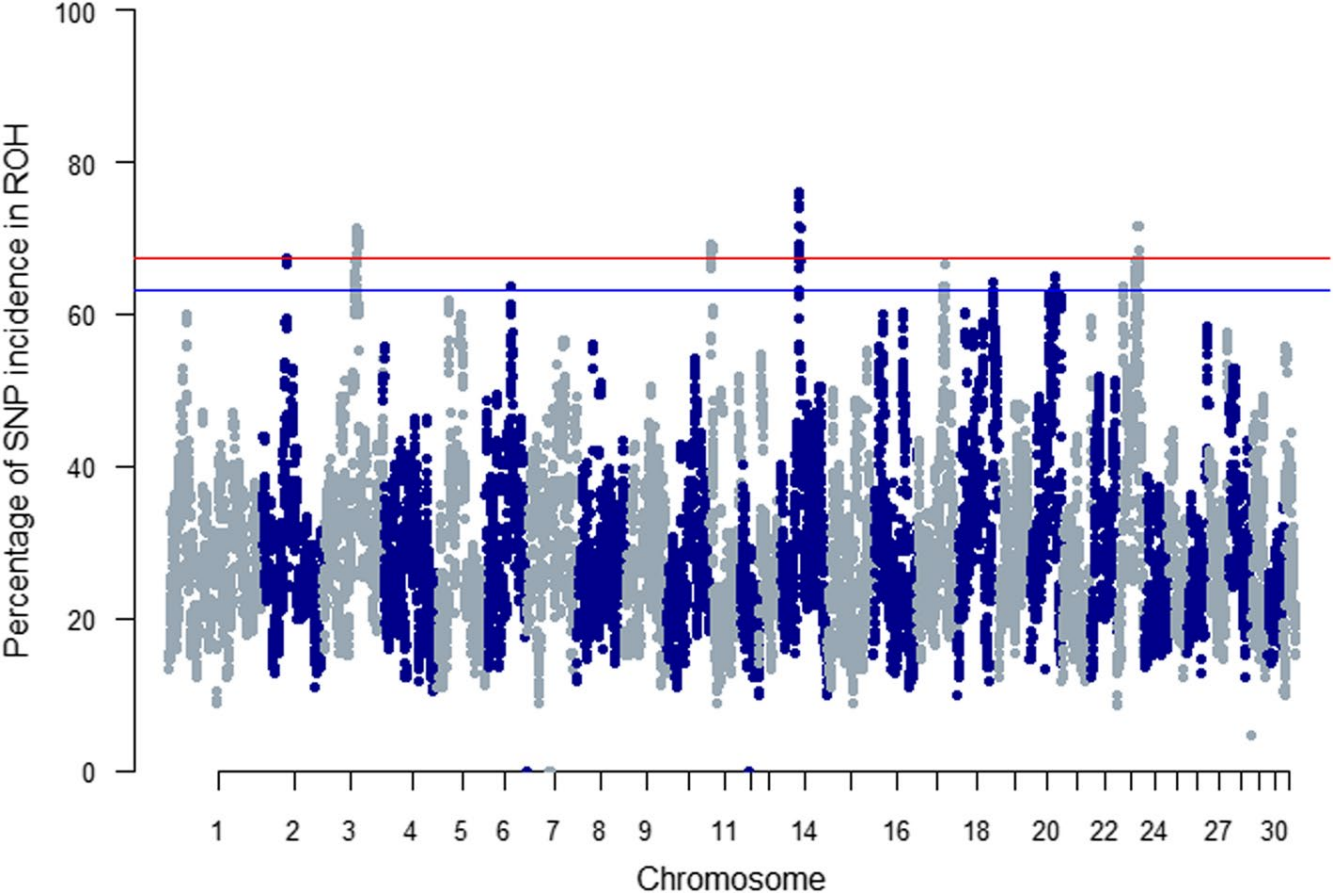
Manhattan plot of incidence of SNPs appearing inside ROH for Sable Island feral horses. Horizontal lines indicate the breed-specific thresholds calculated based on standard normal z-scores generated from SNP-in-ROH incidence in 1 Mbp bins (red), and all SNP-in-ROH incidence (blue), above which ROH islands are indicated

The breed-specific threshold to determine ROH islands in Sable Island horses was an incidence of 67.45 when the binning procedure was used and 63.21 when it was not (Fig. 6; red and blue line, respectively). In Sable Island horses ROH islands were detected on ECA2, ECA3, ECA11, ECA14, and ECA23 following the binning procedure, and additionally on ECA6, ECA17, ECA18 and ECA20 when bins were omitted. While portions of several ROH islands overlapped with those found in domestic breeds, the majority of ROH islands detected in Sable Island horses appeared to be unique to the population. The more conservative analysis (using the binning procedure) revealed some overlap with 33.3% of New Forest Ponies on ECA2 and 36% of Miniature horses on ECA3 (Additional file 2). When bins were omitted, ROH islands overlapped between Sable Island horses and 54.5% of Shires, 33.3% of Newforest Ponies and 64.7% of French Trotters on ECA2; 33.3% of New Forest Ponies and 36% of Miniature Horses on ECA3; 40% of Percherons on ECA14; 44% of Saddlebreds on ECA18; and 66.7% of Exmoor Ponies on ECA23 (see Additional file 2 for corresponding genes, but note that not all overlapping ROH islands contained known genes). A number of genes listed in Additional file 2 are associated with the following traits in horses: joint and hoof health (*ADAMTS*_*3*_ [29]), leopard spotting coat patterns and congenital stationary night blindness (*TRPM1* [30–31]), number of hair whorls on the face (*PTAR1* [32])*,* gait patterns (the “gait keeper” gene *DMRT3* [33–35]), and brown coat colour (*TYRP1* [36–38]). See Additional file 3 for Manhattan plots with ROH thresholds of domestic breeds.

**Fig. 7:**
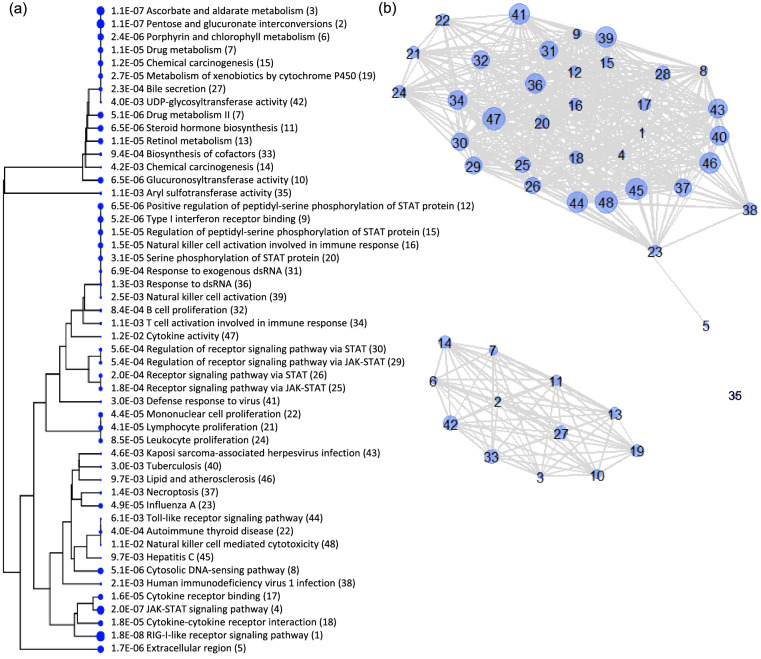
Relationships between pathways of top 50 significant functional categories from GO analysis. Gene ontology (GO) analysis was conducted on all genes contained within ROH islands detected in Sable Island horses without binning. **a** Network tree generated by ShinyGo v0.741 [69] showing relatedness between processes. Branches occur where pathways share common genes, and the size of the dot to the left of each entry corresponds to the level of significance given by the GO analysis. P-values are also provided along with the number given to each node in (**b**). **b** Network matrix of significant pathways showing 3 clusters with shared genes. The size of the node corresponds to the number of genes shared between pathways with larger dots indicating more overlap between pathways. Node labels correspond to the pathways indicated in (**a**)

After searching the regions indicated by the ROH islands analysis, BioMart returned 45 genes in Sable Island ROH islands when binning was used and 264 genes when that constraint was lifted. Notably, some of the smallest ROH islands did not encompass known genes and therefore did not contribute to this list (e.g. the ROH island on ECA23 when using binning). Lists of genes found within ROH islands can be found in Additional file 2. The GO analysis performed to determine if these genes were disproportionately associated with particular functional categories returned a single functional category when binning was used (Nuclear ubiquitin ligase complex, 3 out of 41 possible genes present in the list, p = 0.03). When bins were omitted, the top 50 pathways grouped into 3 clusters and are presented in Fig. 7. One of these clusters included only one significant category (Aryl sulfotransferase activity), while another included 14 significant functional categories representing several processes associated with drug response and metabolism, including bile secretion, chemical carcinogenesis, steroid hormone biosynthesis and metabolism of xenobiotics (Table 3, Fig. 7). The remaining cluster included 35 pathways largely related to immune function, including many related to viral infections and lymphocytes.

**Table 3:** Top 50 Gene ontology (GO) enrichment results for ROH islands in Sable Island feral horses

*P*-value	Genes in list	Total genes	Functional Category
1.76E-08	11	73	RIG-I-like receptor signaling pathway
1.11E-07	7	20	Ascorbate and aldarate metabolism
1.11E-07	7	21	Pentose and glucuronate interconversions
2.01E-07	13	164	JAK-STAT signaling pathway
1.72E-06	37	1741	Extracellular region
2.36E-06	7	34	Porphyrin and chlorophyll metabolism
5.06E-06	8	60	Cytosolic DNA-sensing pathway
5.06E-06	8	60	Drug metabolism
5.21E-06	5	12	Type I interferon receptor binding
6.46E-06	6	25	Glucuronosyltransferase activity
6.46E-06	5	13	Positive regulation of peptidyl-serine phosphorylation of STAT protein
6.46E-06	8	65	Steroid hormone biosynthesis
1.12E-05	7	47	Drug metabolism
1.12E-05	8	71	Retinol metabolism
1.19E-05	7	48	Chemical carcinogenesis
1.48E-05	5	16	Natural killer cell activation involved in immune response
1.48E-05	5	16	Regulation of peptidyl-serine phosphorylation of STAT protein
1.56E-05	12	221	Cytokine receptor binding
1.80E-05	13	271	Cytokine-cytokine receptor interaction
2.67E-05	7	56	Metabolism of xenobiotics by cytochrome P450
3.14E-05	5	19	Serine phosphorylation of STAT protein
4.12E-05	11	201	Lymphocyte proliferation
4.35E-05	11	203	Mononuclear cell proliferation
4.93E-05	10	164	Influenza A
8.52E-05	11	219	Leukocyte proliferation
1.83E-04	8	110	Receptor signaling pathway via JAK-STAT
2.02E-04	8	112	Receptor signaling pathway via STAT
2.25E-04	7	80	Bile secretion
4.03E-04	6	57	Autoimmune thyroid disease
5.38E-04	7	92	Regulation of receptor signaling pathway via JAK-STAT
5.60E-04	7	93	Regulation of receptor signaling pathway via STAT
6.95E-04	5	37	Response to exogenous dsRNA
8.41E-04	6	66	B cell proliferation
9.39E-04	8	142	Biosynthesis of cofactors
1.12E-03	6	70	T cell activation involved in immune response
1.13E-03	3	7	Aryl sulfotransferase activity
1.28E-03	5	43	Response to dsRNA
1.45E-03	8	153	Necroptosis
2.05E-03	9	210	Human immunodeficiency virus 1 infection
2.50E-03	5	50	Natural killer cell activation
2.98E-03	8	171	Tuberculosis
3.03E-03	8	172	Defense response to virus
3.95E-03	7	132	UDP-glycosyltransferase activity
4.15E-03	8	181	Chemical carcinogenesis
4.56E-03	8	184	Kaposi sarcoma-associated herpesvirus infection
6.09E-03	6	99	Toll-like receptor signaling pathway
9.71E-03	7	155	Hepatitis C
9.71E-03	8	207	Lipid and atherosclerosis
1.05E-02	7	158	Cytokine activity
1.05E-02	6	111	Natural killer cell mediated cytotoxicity

Generated using data when no binning procedure was used. The number of genes present in the associated list (Additional file 2) compared to the total number of genes in the horse genome, as well as corresponding P-values, are shown for each functional category. These functional categories are disproportionately represented by the genes found within ROH islands in Sable Island horses

## Discussion

In this study we sought to understand whether patterns of inbreeding differed between Sable Island horses and domestic breeds, if ROH islands found in Sable Island horses were unique to this population, and if genes located within ROH islands could provide insight into the nature of population divergence.

Sable Island horses exhibited the largest average number of ROH of all horse populations studied, with less variation in abundance than their domestic counterparts. This is unsurprising given the wide variety of domestic breeds studied and the small size of the Sable Island population. For context, two of the domestic breeds are listed as “rare” with no population estimate provided while the remaining populations ranged from approximately 2000 to millions of individuals, each with unique population histories and contemporary management practices associated with them [39], which is likely to result in a wide range of ROH characteristics. In contrast, the Sable Island population typically ranges from 250 to 550 individuals but has been recorded as low as 133 [27, 40]. Additionally, the population experiences frequent crashes following harsh winters and has been genetically isolated since 1935 [27]. Effective population size has been estimated at approximately 48 individuals [28], severely limiting the number of haplotypes that can be passed on, and a large number of ROH spread across the genome is likely to occur as a result [41].

ROH were generally more abundant on larger chromosomes and less so on shorter chromosomes with the exception of the relatively low number of ROH present on ECA12 and ECA13 compared to their size. More genetic material provides more chances for ROH presence, but recombination rate likely plays an important role in the ROH distribution. Some research has shown that increased recombination rates tend to occur on shorter chromosomes [42]. Higher recombination rates lead to shorter ROH, increasing the likelihood they be undetected when using a limited number of SNPs, but research in Soay sheep revealed that recombination rate accounts for only a small portion of variation in detected ROH density, particularly when short ROH were considered [22]. For horses, mean recombination rate has been reported to be similar across most chromosomes, with no clear correlation between chromosome length and average recombination rate or number of recombination hotspots [43]. In addition, a particularly high mean recombination rate on ECA12 has been published (2.13 cM/Mb vs an overall average of 1.24 cM/Mb) [43], which could account for the low number of ROH found on that chromosome in the present study. This does not explain the results on ECA13, but SNP density might. The SNPs in the dataset used here had representation from all autosomes, but the number of SNPs on each chromosome was not proportional to chromosome length in all cases with ECA12 and ECA13, as well as ECA26, being clear outliers (Additional file 4). It is unclear why these chromosomes have lower SNP densities, but it may be related to the initial goals and methods used during the creation of horse SNP chips [44]. Caution should be used when applying recombination rates calculated for domestic breeds to the feral population owing to the notable between-breed differences in recombination rates and hot- and cold- spots found in a variety of horse breeds [43], particularly in light of lower than expected impacts of recombination rate on ROH in other species [22]. Producing a population-specific linkage map for Sable Island horses would allow for a better understanding of the relationship between ROH and recombination rate, and whether the signatures of selection found here correlate with recombination coldspots, for example, as they do in other breeds [43].

The relative proportion of ROH lengths within populations differed markedly between Sable Island horses and their domestic counterparts. In particular, Sable Island horses had the smallest proportion of runs 0-2 Mb in length and the largest proportion in the 4-8 Mb length class, suggesting shorter coalescence time than in their domestic counterparts. The relationship between domestication and ROH length is context dependent and the comparison of ROH in wild or feral versus domestic populations of livestock has previously yielded mixed results. For example, a study of wild boars and domestic pigs in Romania revealed much longer ROH, a sign of recent inbreeding and population bottlenecks, in wild as compared to domestic populations [20]. The authors attribute this pattern to overhunting and/or infectious disease outbreak in wild boars [20]. In contrast, a similar study in the Iberian Peninsula found that domestic pig populations had more signs of recent inbreeding while their wild counterparts had much shorter, albeit abundant, ROH indicating past population bottlenecks but a lack of recent inbreeding [21]. The Sable Island horse results indicate that historical population bottlenecks and inbreeding happened slightly more recently than in their domestic counterparts, but the relative absence of very long (>16 Mb) ROH demonstrates a lack of contemporary mating among closely related individuals. This may be the case if inbreeding avoidance mechanisms are intact in the population. Inbreeding avoidance behaviour has been observed in other feral horse populations [45–47], and dispersal patterns in juvenile Sable Island horses are consistent with inbreeding avoidance [48]. However, consanguineous matings may be underestimated by our results if they result in non-viable offspring, or highly inbred individuals die young and are not detected for sampling. This pattern has been seen in other ungulate populations; for example, research in Soay sheep has shown dramatic decreases in survival rates of highly inbred lambs [22].

Looking at inbreeding coefficients specifically, F_ROH_ was highest in Sable Island horses, but several domestic breeds had similar values. Variation in F_ROH_ seen in domestic horses was largely in agreement with similar inbreeding estimates derived from the same data by Petersen et al. [49] and follow expected trends based on the age and size of each breed, as well as management and breeding practices [49]. Minor differences in F_ROH_ values compared to previously published inbreeding coefficients can likely be explained by differences in filtering for linkage disequilibrium and the specific inbreeding metrics being used. The elevated F_ROH_ in Sable Island horses is consistent with the population’s small size, genetic isolation, and lack of management. In fact, it was surprising that F_ROH_ was not even more elevated compared to domestic breeds, but the tight correlation between F_ROH_ and F_IS_ values in this population supports F_ROH_ as an accurate representation of consanguinity rather than an unexpected side effect of population structure [50]. When F_ROH_ is equal to F_IS_ it indicates that all excess homozygosity is accounted for by ROH [50]. In contrast, when F_ROH_ is greater than F_IS_ as in several domestic horses shown here, it suggests small effective population size (N_e_) or founder effects limiting the number of available haplotypes (therefore increasing ROH presence) despite random mating (F_IS_ = 0) or inbreeding avoidance (F_IS_ < 0) in the most recent generation(s) [50].

Although it should not generally be necessary in domestic populations due to management practices, inbreeding avoidance likely occurs in Sable Island horses while elevated inbreeding estimates in domestic breeds are likely due to founder effects and early historical population bottlenecks (as supported by the abundant short ROH found in domestic breeds in this study as well as the relationship between F_ROH_ and F_IS_). These factors may combine to produce comparable overall inbreeding metrics between feral and domestic populations. The ways in which F_ROH_ was expressed in the genome varied between populations, and closely reflected population history. Sable Island horses tended to have high incidence of ROH on most but not all chromosomes which does not necessarily reflect the expected results of inbreeding alone (i.e. random distribution across the genome). Uneven distribution of ROH in the genome is to be expected based on differences in recombination rates of various genomic regions and other stochastic processes such as genetic drift, but is also expected in the case of selection (either natural or artificial [14]). Indeed, the chromosomes with the highest F_ROH_ were also those on which most ROH islands were found in Sable Island horses.

ROH islands were found in all horse breeds studied, with between five and nine islands detected in the Sable Island genome, depending on the analysis. The results from domestic breeds were generally well aligned with those recently published in a publicly available ROH island repository [15]; in some cases, islands found previously were not detected here and vice versa, but these discrepancies can likely be explained by differences in SNP filtering protocols and ROH parameters. In domestic breeds, it is expected that the majority of these signals be the result of artificial selection, and the results published here and elsewhere support this. If, for example, this analysis was detecting signatures of selection that occurred prior to the domestication of the horse, the same signatures should be visible in all or most modern breeds but this is not the case. The presence of relatively unique signatures of selection is consistent with previous studies in horses which have shown breed differentiation and associations with breed-specific and performance related traits (e.g. [14, 49, 51–59]). The extent of the selective breeding that occurred in the Sable Island population was the intentional removal of “coloured” horses (e.g. paints and greys) from the island, which could perhaps explain the presence of the brown coat colour gene [36–38] appearing in ROH islands. Simultaneously, select mares and stallions were introduced into the population between 1801 and 1940 [27] and young horses were removed from the island to be sold in Halifax with unknown and likely variable impacts on population level genetic diversity [24, 26]. While it remains unclear if the rare instances of ROH island overlap between Sable Island horses and domestic breeds are indicative of contributions of these breeds to the feral population, similar contemporary selection pressures, or chance, these signatures in Sable Island horses appeared relatively unique compared to the other breeds. When overlap did occur, it often only encompassed a single SNP, and in no case was the overlap complete. This suggests that the Sable Island population has experienced unique divergence since isolation from domestic breeds, possibly in response to selection. However, small effective population size (N_e_), which is likely to occur in small isolated populations in the wild as well as during artificial selection in domestic species, contributes to an increase in genetic drift [41]. Along with artificial or natural selection, genetic drift is expected to increase the occurrence of long ROH and spurious ROH islands, making it difficult or impossible to distinguish the precise cause of such genomic signatures [41].

Totals of 42 and 264 genes were identified in Sable Island ROH Islands, depending on the island detection threshold used. The more conservative analysis resulted in a small number of genes and only one significant functional category in the GO analysis. However, when a less conservative threshold was used, GO analysis revealed an overrepresentation of genes associated with immune function, metabolism and development. While the results could be due to drift, they are nonetheless consistent with the selective pressures one would expect for a population which exists in a harsh environment with no human intervention. For example, Sable Island horses experience extreme fluctuations in the quality and availability of both forage and water, with food scarcity being common in winter [40], and horses are frequently observed eating beach pea (*Lathyrus maritimus* L.) which may contain toxic compounds [60]. Additionally, parasite levels on the island are elevated [61] and individual parasite load is correlated with variation in body condition [62]. Although several domestic breeds exist in sandy conditions, Sable Island horses do not benefit from hoof or dental maintenance to combat associated issues, and their only shelter from the elements are sand dunes. The genes within ROH islands detected here may confer a fitness advantage that allows horses to survive and reproduce despite these challenges if their presence in ROH islands is a result of selection. For example, selection for bile secretion genes may be associated with the ability to withstand repeated periods of near starvation as forage availability fluctuates seasonally and from year to year. Different genes associated with bile secretion were found in a similar analysis of Arabian horses [52], which may support a connection between selection for bile secretion genes and barren sandy landscapes. Conversely, if some or most of the genes in ROH islands are present due to genetic drift or genetic hitchhiking, the alleles present could have neutral or detrimental impacts on fitness. The SNPs used in this analysis do not necessarily equate to different coding region variants, so further work is needed to better understand the fitness effects, if any, of elevated homozygosity in these regions. Regardless, the possibility that Sable Island horses constitute a genetic reservoir of various aspects of immune function and metabolism due to the unique selective pressures they face represents an interesting avenue for future exploration. Additionally, further work is needed to understand the impact on the Sable Island horse population of those genes which were detected in ROH islands and are associated with specific traits in horses (i.e. coat colour and growth patterns [30–32*,* 36–38], variations in gait [33–35], and joint, hoof [29] and ocular health [30, 31]) but did not strongly impact the results of GO analysis.

## Conclusions

Here we applied ROH analyses in a feral horse population of conservation concern to provide insight into its genetic health and divergence from domestic breeds. Based on ROH length, abundance and their related inbreeding coefficient (F_ROH_), Sable Island horses appear to be more inbred than their domestic counterparts. Furthermore, ROH length patterns suggest founder effects and population bottlenecks have occurred more recently in Sable Island horses than in their domestic counterparts, but mating between very close relatives remains rare. Several ROH islands typical of selection were found in Sable Island horses and these regions were enriched for genes involved in metabolism and immune function. Future work should focus on determining if ROH islands could be explained by genetic drift, the effects of inbreeding on fitness (inbreeding depression), and the direct impacts of genes located in ROH islands.

## Methods

### Study area and sampling

Sable Island National Park Reserve (Fig. 1) is a long, narrow sand bar (approximately 49 km in length and 1.25 km at its widest point), located approximately 275 km southeast of Halifax, Nova Scotia along the continental shelf of the Atlantic Ocean [48]. The island is characterized by bare and vegetated sand dunes up to 30 meters in elevation, large grassy planes, low heathlands and wide sandy beaches. Access to the island is controlled, and human activity is limited. A small (n ≈ 250 – 550; [40]) unmanaged population of feral horses has existed on the island since the mid-1700s, and is currently the only species of land mammal inhabiting the island [24]. Since 2008, census data has been collected via systematic ground surveys as part of an ongoing individual-based study [48]. Population census includes extensive photography of any markings or distinguishing characteristics in order to identify individuals. From 2008 to 2012, tail hair samples used for genetic analysis were opportunistically sampled from known individuals when it was deemed safe to do so by observers. This method was discontinued in 2013 when Sable Island became a national park and new regulations surrounding wildlife interactions were put in place. From 2014 to 2016, opportunistic saliva samples were taken by swabbing vegetation that had been dropped from the mouths of horses or had been grazed leaving visible saliva on grass shoots. Tissue samples in the form of ear snips were taken when horses were found dead. Although carcasses are often difficult to identify, in 2015 a known individual died during the field season and a fresh tissue sample was taken and used in this analysis. Sampling and genotyping was carried out under University of Saskatchewan Animal Care Protocol 20090032, University of Calgary Animal Care Protocol AC18-0078, and research permits granted by Parks Canada (SINP-2017-24036 and SINP-2021-38998).

### DNA extraction, genotyping and filtering

DNA samples from 218 Sable Island horses were extracted from hair roots using Qiagen’s User-Developed Isolation of genomic DNA from nails and hair Protocol (QA05 Jul-10) and the QIAamp DNA Micro Kit, from saliva using the DNA PERFORMAgene PG-100 kit (DNA Genotek Inc., Ottawa, Canada) and the recommended protocol, and from tissue using Qiagen’s DNeasy Blood & Tissue Kit and the recommended protocol. DNA was then eluted in molecular grade water and quantified using a Qubit fluorometer with the dsDNA Broad Range Assay Kit (Invitrogen, United States) before being dried down and shipped to Geneseek/Neogen (Lincoln, United States) for genotyping on Illumina equine SNP arrays (400 ng per sample). Ninety-eight and 120 samples were genotyped on the GGP65 and GGP65Plus arrays, respectively. These data were combined with those from 795 horses from 33 domestic breeds available from [49].

Illumina equine SNP arrays were originally developed using the second version of the horse genome assembly (EquCab2 [63]) but a newer genome assembly has since become available (EquCab3 [64]). In this study, we only retained SNPs which mapped to a unique EcuCab3 position when using both the approach of [65] and the NCBI Genome Remapping Service (https://www.ncbi.nlm.nih.gov/genome/tools/remap), and used corresponding EquCab3 positions in all analyses. We limited analyses to the 41 944 SNPs that were genotyped on all arrays in order for results to be comparable across samples.

Genotype data were formatted and filtered using R and plink v1.90 [66]. After excluding SNPs on sex chromosomes, individuals and SNPs with genotyping rate < 90%, and SNPs with minor allele frequency of < 0.001, 41 035 SNPs and 935 individuals were retained. Of those, 212 were Sable Island feral horses and 723 were from domestic breeds (n = 14 – 43 per breed). None of the saliva samples passed quality control. The age, history, location and population size of all domestic breeds used was highly variable, and details can be found in [49].

### Runs of homozygosity and inbreeding

Runs of homozygosity were calculated for all 31 autosomes using the consecutive runs function in the detectRUNS package in R [67]. In order to be included, runs had to contain a minimum of 30 consecutive SNPs, a maximum gap of 1 megabase (Mb), and a maximum of 2 missing SNPs. The analysis was repeated with a maximum number of heterozygous SNPs allowed within a run at 1, 2 and 3 to account for possible genotyping errors. Results were qualitatively similar for all 3 levels of heterozygosity, so only the most stringent analysis was used subsequently. To explore the relative length of ROH, five length classes were used: 0-2 Mb, 2-4 Mb, 4-6 Mb, 8-16 Mb, and >16 Mb. Overall and chromosome specific ROH-based inbreeding coefficients (F_ROH_) were calculated for all individuals as the proportion of the genome contained within runs versus the length of the genome or chromosome, respectively. To explore the relative contribution of various run lengths to inbreeding, F_ROH_ was also calculated based on the following run length classes: >0 Mb, >2 Mb, >4 Mb, >8 Mb, and >16 Mb. Additionally, F_IS_ was calculated using the --het function in plink v1.90 [66] and plotted against corresponding genome-wide F_ROH_ values to determine the relationship between ROH and inbreeding in the current generation due to non-random mating [50].

### ROH islands and signatures of selection

The incidence of each SNP occurring within a run was calculated for each population with the “snpInsideRuns” function in the detectRUNS package in R [67]. As per [15], ROH islands were defined as regions where the p-value (based on normal z-scores) for SNP incidence was above a population-specific threshold. In order to determine these thresholds, a binning procedure was conducted to account for variation in SNP density throughout the genome [4]. The genome was divided into 1Mb bins and only the SNP with the highest incidence in each bin was used for further calculations. Normal z-scores and corresponding p-values were calculated and SNPs with p>0.999 were considered to surpass the population-specific threshold and form the basis of ROH islands [4, 5]. Further, population-specific thresholds were held to a minimum of 30% and a maximum of 80% as per [15] to ensure populations in which all SNPs had very high ROH incidence did not result in erroneous islands, and that islands were not missed in cases when no SNPs reached the *p*>0.999 cutoff. This analysis was also repeated without the binning process so that all SNPs could be considered and results compared.

For Sable Island horses, genome regions encompassed by ROH islands were used to extract gene names and functions using Ensembl BioMart (release 105 [68]). The positions of the first and last consecutive SNP above the ROH island threshold were used as the boundaries within which genes were searched. A gene ontology (GO) enrichment analysis was then performed on the resulting list of genes using ShinyGO v0.741 [69] with a p-value cutoff of 0.05 and the top 50 pathways shown. GO analysis returns functional categories of genes and biological pathways that occur more than would be expected by chance based on the abundance of genes within each functional category in the genome.

## Supplementary Information

**Additional file 1.** Excel spreadsheet containing a table too wide for A4. Mean per-chromosome ROH-based inbreeding coefficients (F_ROH_) by horse population. Average inbreeding coefficients derived by dividing the length of each chromosome present within ROH by the total length of the corresponding chromosome for individuals from 33 domestic horse breeds and Sable Island feral horses.

**Additional file 2.** ROH island genes in Sable Island horses. List of genes which are present in ROH islands of Sable Island feral horses. All entries were found when all available SNPs were used in the analysis while bolded entries were also found when the binning procedure was used. The Domestic Breed column indicates which of the domestic horse populations studied here have the same gene present in ROH islands, with the percentage of individuals within those breeds which exhibited ROH islands in those areas indicated in parentheses.

**Additional file 3.** ROH islands of domestic horse breeds. Manhattan plots of incidence of SNPs appearing inside ROH for each of the 33 domestic horse breeds in the analysis. Abbreviated breed names and sample sizes are indicated in the top left corner of each plot. Horizontal lines indicate the breed-specific thresholds calculated based on standard normal z-scores generated from SNP-in-ROH incidence in 1 Mbp bins (red), and all SNP-in-ROH incidence (blue), above which ROH islands are indicated. In instances where only one line is visible, the values for the two thresholds are identical.

**Additional file 4.** SNP density (SNP/Mb) per chromosome. Density of SNPs on each chromosome as calculated by the total number of SNPs used in the final dataset per chromosome divided by chromosome length in Mb.

## Data Availability

The domestic horse dataset analysed in the current study is available in the Animal Genome repository (https://www.animalgenome.org/repository/pub/UMN2012.1130/). Sable Island genotypes are available from the corresponding authors on reasonable request.

## Abbreviations

ROH: runs of homozygosity
AKTK: Akhal Teke horse
AND: Andalusian horse
ARR: Arabian horse
BEL: Belgian horse
CLYD: Clydesdale horse
CSP: Caspian horse
EXMR: Exmoor Pony
FELL: Fell Pony
FINN: Finnhorse
FM: Franches-Montagnes horse
FT: French Trotter horse
HAN: Hanovarian horse
ICE: Icelandic horse
MINI: Miniature horse
MNGP: Mangalara Paulista horse
MON: Mongolian horse
MOR: Morgan horse
NFST: New Forest Pony
NORF: Norwegian Fjord horse
NSWE: North Swedish horse
PERC: Percheron horse
PERU: Peruvian Paso horse
PRPF: Puerto Rican Paso Fina horse
PT: Paint horse
QH: Quarter Horse
SB: Saddlebred horse
SHET: Shetland pony
SHR: Shire horse
SI: Sable Island feral horse
STBD: Standardbred horse
SZWB: Swiss Warmblood horse
TBUK: European Thoroughbred horse
TBUS: American Thoroughbred horse
TUVA: Tuva horse
