# Supplementary material for: Runs of homozygosity in Sable Island feral horses reveal the genomic consequences of inbreeding and divergence from domestic breeds
Source: BMC Genomics. 2022 Jul 12;23:501. doi: 10.1186/s12864-022-08729-9 (PMC9275264; doi:10.1186/s12864-022-08729-9)
Supplement: Supplementary file 2 — Additional file 2. ROH island genes in Sable Island horses. List of genes which are present in ROH islands of Sable Island feral horses. All entries were found when all available SNPs were used in the analysis while bolded entries were also found when the binning procedure was used. The Domestic Breed column indicates which of the domestic horse populations studied here have the same gene present in ROH islands, with the percentage of individuals within those breeds which exhibited ROH islands in those areas indicated in parentheses. [file 12864_2022_8729_MOESM2_ESM.docx]

| **Chromosome** | **Gene start (bp)** | **Gene end (bp)** | **Gene name** | **Gene stable ID** | **Domestic Breed** |
| --- | --- | --- | --- | --- | --- |
| 2 | 47448624 | 47471555 | TTC34 | ENSECAG00000020233 | Shire (54.5%), French Trotter (64.7%), NewForest Pony (33.3%) |
| 2 | 47480784 | 47514465 | MMEL1 | ENSECAG00000020732 | NewForest Pony (33.3%) |
| 2 | 47537791 | 47560697 | TNFRSF14 | ENSECAG00000008751 | NewForest Pony (33.3%) |
| 2 | 47572171 | 47609543 |  | ENSECAG00000030194 | NewForest Pony (33.3%) |
| 2 | 47624193 | 47624858 | HES5 | ENSECAG00000036474 | NewForest Pony (33.3%) |
| 2 | 47627626 | 47645561 | PANK4 | ENSECAG00000035260 | NewForest Pony (33.3%) |
| 2 | 47735065 | 47740468 | PEX10 | ENSECAG00000009743 | NewForest Pony (33.3%) |
| 2 | 47753490 | 47754599 |  | ENSECAG00000040937 | NewForest Pony (33.3%) |
| 2 | 47754718 | 47782439 | MORN1 | ENSECAG00000043532 | NewForest Pony (33.3%) |
| 2 | 47907098 | 47909092 |  | ENSECAG00000039334 | NewForest Pony (33.3%) |
| 2 | 47932890 | 47942149 | FAAP20 | ENSECAG00000014263 | NewForest Pony (33.3%) |
| 2 | 48111157 | 48173507 | CFAP74 | ENSECAG00000020515 | NewForest Pony (33.3%) |
| 2 | 48175846 | 48177698 | TMEM52 | ENSECAG00000032870 | NewForest Pony (33.3%) |
| **2** | **48216779** | **48276769** | **GNB1** | **ENSECAG00000021999** | **NewForest Pony (33.3%)** |
| 3 | 62836406 | 62836512 | U6 | ENSECAG00000026021 | NewForest Pony (33.3%) |
| 3 | 62875070 | 62915915 | BTC | ENSECAG00000022505 | NewForest Pony (33.3%) |
| 3 | 62933998 | 62939992 |  | ENSECAG00000040028 | NewForest Pony (33.3%) |
| 3 | 63027761 | 63069300 |  | ENSECAG00000030543 |  |
| 3 | 63661747 | 63673088 |  | ENSECAG00000043341 |  |
| 3 | 63728490 | 63758180 |  | ENSECAG00000036926 |  |
| 3 | 63759518 | 63762163 |  | ENSECAG00000039527 |  |
| 3 | 63817410 | 63867349 | RASSF6 | ENSECAG00000019471 |  |
| 3 | 64119696 | 64282337 | ANKRD17 | ENSECAG00000007125 |  |
| 3 | 64288729 | 64299360 | COX18 | ENSECAG00000017173 |  |
| 3 | 64412562 | 64434509 |  | ENSECAG00000043736 |  |
| 3 | 64640211 | 64912160 | ADAMTS3 | ENSECAG00000019061 |  |
| 3 | 65243429 | 65273923 | GC | ENSECAG00000010481 |  |
| 3 | 65950263 | 65962892 | GRSF1 | ENSECAG00000024208 |  |
| 3 | 66086164 | 66096563 | JCHAIN | ENSECAG00000020041 |  |
| 3 | 66620215 | 66638866 |  | ENSECAG00000034147 |  |
| 3 | 66736819 | 66743224 | CSN2 | ENSECAG00000009837 |  |
| 3 | 66832214 | 66971453 | SULT1E1 | ENSECAG00000034051 |  |
| 3 | 67001370 | 67014011 |  | ENSECAG00000028627 |  |
| 3 | 67033564 | 67056462 | SULT1B1 | ENSECAG00000000863 |  |
| 3 | 67107705 | 67171003 | UGT2A1 | ENSECAG00000037095 |  |
| 3 | 67367454 | 67390014 |  | ENSECAG00000040845 |  |
| 3 | 67441267 | 67483197 |  | ENSECAG00000028064 |  |
| 3 | 67529185 | 67555286 |  | ENSECAG00000032809 |  |
| 3 | 67605505 | 67652422 |  | ENSECAG00000033324 |  |
| 3 | 67677901 | 67691727 |  | ENSECAG00000036717 |  |
| 3 | 67728336 | 67790931 |  | ENSECAG00000040172 |  |
| 3 | 67824146 | 67918805 |  | ENSECAG00000020628 |  |
| 3 | 68003629 | 68028230 |  | ENSECAG00000029516 |  |
| 3 | 68154428 | 68173911 |  | ENSECAG00000017275 |  |
| 3 | 68241838 | 68254132 |  | ENSECAG00000018165 |  |
| 3 | 68285866 | 68286765 |  | ENSECAG00000033060 |  |
| 3 | 68415693 | 68415793 | U6 | ENSECAG00000025570 |  |
| 3 | 68435308 | 68478065 | TMPRSS11E | ENSECAG00000023819 |  |
| 3 | 68516940 | 68532870 | TMPRSS11B | ENSECAG00000024160 |  |
| 3 | 68542933 | 68561982 |  | ENSECAG00000024588 |  |
| 3 | 68620064 | 68700016 | TMPRSS11F | ENSECAG00000006671 |  |
| 3 | 68709962 | 68748851 |  | ENSECAG00000008590 |  |
| **3** | **68709962** | **68748851** |  | **ENSECAG00000008590** |  |
| **3** | **68766212** | **68815260** | **TMPRSS11A** | **ENSECAG00000009414** |  |
| **3** | **68833561** | **68888176** | **TMPRSS11D** | **ENSECAG00000010705** |  |
| **3** | **68904248** | **68962967** |  | **ENSECAG00000011052** |  |
| **3** | **68976214** | **68997102** | **GNRHR** | **ENSECAG00000005763** |  |
| **3** | **69018543** | **69099948** | **UBA6** | **ENSECAG00000012273** | **Miniature Horse (36%)** |
| **3** | **69158288** | **69235012** | **CENPC** | **ENSECAG00000018267** | **Miniature Horse (36%)** |
| **3** | **69392271** | **69392399** |  | **ENSECAG00000027585** | **Miniature Horse (36%)** |
| **3** | **69455329** | **69517219** |  | **ENSECAG00000036677** | **Miniature Horse (36%)** |
| **3** | **69648516** | **69655144** |  | **ENSECAG00000035585** | **Miniature Horse (36%)** |
| **3** | **69862939** | **69866545** |  | **ENSECAG00000032914** | **Miniature Horse (36%)** |
| **3** | **69908420** | **69943310** |  | **ENSECAG00000042191** | **Miniature Horse (36%)** |
| **3** | **70757826** | **71114534** | **EPHA5** | **ENSECAG00000000530** |  |
| 11 | 1812146 | 1978077 | SLC38A10 | ENSECAG00000013118 |  |
| 11 | 1875261 | 1885591 | TEPSIN | ENSECAG00000024720 |  |
| 11 | 1900486 | 1918763 | CEP131 | ENSECAG00000007579 |  |
| 11 | 1937694 | 1976989 | AATK | ENSECAG00000021405 | Percheron (40%) |
| 11 | 2477706 | 2478734 |  | ENSECAG00000032813 |  |
| 11 | 2551544 | 2558116 | NPTX1 | ENSECAG00000011663 |  |
| 11 | 2560639 | 2560967 |  | ENSECAG00000035602 |  |
| 11 | 2758831 | 2765286 | SGSH | ENSECAG00000004844 |  |
| 11 | 2808576 | 2824252 | EIF4A3 | ENSECAG00000015925 |  |
| **11** | **2894289** | **2999422** | **TBC1D16** | **ENSECAG00000018094** |  |
| **11** | **3052867** | **3059517** | **CBX4** | **ENSECAG00000001181** |  |
| **11** | **3093600** | **3096920** | **CBX8** | **ENSECAG00000019253** |  |
| **11** | **3354336** | **3684855** | **RBFOX3** | **ENSECAG00000023811** |  |
| **11** | **3701945** | **3705665** |  | **ENSECAG00000023129** |  |
| **11** | **3745146** | **3758743** | **CANT1** | **ENSECAG00000023633** |  |
| **11** | **3769018** | **3779810** | **LGALS3BP** | **ENSECAG00000033385** |  |
| **11** | **3793239** | **3793963** |  | **ENSECAG00000036395** |  |
| **11** | **3811598** | **3858411** | **TIMP2** | **ENSECAG00000011834** |  |
| **11** | **3872483** | **3971069** | **USP36** | **ENSECAG00000016116** |  |
| **11** | **3917948** | **3999258** | **CYTH1** | **ENSECAG00000024699** |  |
| **11** | **3987006** | **3987108** | **U6** | **ENSECAG00000027091** |  |
| **11** | **4041521** | **4154288** | **DNAH17** | **ENSECAG00000002831** |  |
| 11 | 4205582 | 4206271 | SOCS3 | ENSECAG00000001249 |  |
| 11 | 4276269 | 4281162 |  | ENSECAG00000034493 |  |
| 11 | 4283132 | 4288706 |  | ENSECAG00000007112 |  |
| 11 | 4338733 | 4350527 | TK1 | ENSECAG00000016173 |  |
| 11 | 4382786 | 4398939 | TMC6 | ENSECAG00000020115 |  |
| 11 | 4578951 | 4580885 |  | ENSECAG00000037437 |  |
| 11 | 4587056 | 4608052 |  | ENSECAG00000043475 |  |
| 11 | 4669989 | 4672474 |  | ENSECAG00000038914 |  |
| 14 | 36052197 | 36239564 | NRG2 | ENSECAG00000013341 |  |
| 14 | 36509803 | 36514617 | STING1 | ENSECAG00000017396 |  |
| 14 | 36525923 | 36533650 |  | ENSECAG00000017697 |  |
| 14 | 36547581 | 36565275 | DNAJC18 | ENSECAG00000018646 | Exmoor Pony (66.7%) |
| 14 | 36572325 | 36577619 | SPATA24 | ENSECAG00000020376 |  |
| 14 | 36579552 | 36582611 | PROB1 | ENSECAG00000005235 |  |
| 14 | 36584849 | 36586698 | MZB1 | ENSECAG00000020503 |  |
| 14 | 36589472 | 36598399 | SLC23A1 | ENSECAG00000021087 |  |
| 14 | 36706241 | 36708787 |  | ENSECAG00000041621 |  |
| 14 | 36740660 | 36929510 | SIL1 | ENSECAG00000019628 |  |
| **14** | **37005563** | **37007458** | **LRRTM2** | **ENSECAG00000007331** |  |
| **14** | **37256810** | **37272259** | **HSPA9** | **ENSECAG00000007513** |  |
| **14** | **37267107** | **37267181** | **SNORD63** | **ENSECAG00000025479** |  |
| **14** | **37269015** | **37269075** | **SNORD63** | **ENSECAG00000027617** |  |
| **14** | **37283086** | **37310442** | **ETF1** | **ENSECAG00000013041** |  |
| **14** | **37438511** | **37461840** | **CDC25C** | **ENSECAG00000003029** |  |
| **14** | **37467809** | **37483774** | **GFRA3** | **ENSECAG00000009357** |  |
| **14** | **37513167** | **37534506** | **CDC23** | **ENSECAG00000011522** |  |
| **14** | **37544375** | **37574250** | **BRD8** | **ENSECAG00000022484** |  |
| **14** | **37575466** | **37604136** | **NME5** | **ENSECAG00000014544** |  |
| **14** | **37691605** | **37772721** | **FAM13B** | **ENSECAG00000017901** |  |
| **14** | **37917883** | **37918788** | **HNRNPA0** | **ENSECAG00000032164** |  |
| **14** | **37939773** | **38046632** | **KLHL3** | **ENSECAG00000022073** |  |
| **14** | **38023400** | **38023483** | **MIR874** | **ENSECAG00000026540** |  |
| **14** | **38082406** | **38095123** |  | **ENSECAG00000043063** |  |
| **14** | **38146566** | **38148722** |  | **ENSECAG00000041103** |  |
| **14** | **38238040** | **38616376** | **SPOCK1** | **ENSECAG00000007511** |  |
| **14** | **39058579** | **39190034** | **TRPC7** | **ENSECAG00000007659** |  |
| 14 | 39210493 | 39210804 | SMIM32 | ENSECAG00000034812 |  |
| 17 | 53572440 | 53578864 |  | ENSECAG00000029418 |  |
| 17 | 53866819 | 53897381 |  | ENSECAG00000031659 |  |
| 17 | 53959631 | 54025906 |  | ENSECAG00000040120 |  |
| 20 | 46709130 | 46711095 |  | ENSECAG00000027961 |  |
| 20 | 46819596 | 46946512 | CD2AP | ENSECAG00000018037 |  |
| 20 | 46974236 | 47013610 | ADGRF2 | ENSECAG00000023950 |  |
| 20 | 47015115 | 47039028 | ADGRF4 | ENSECAG00000024011 |  |
| 20 | 47099217 | 47146594 | OPN5 | ENSECAG00000024718 |  |
| 20 | 47745827 | 47786925 |  | ENSECAG00000035292 |  |
| 20 | 48494909 | 48508022 | CENPQ | ENSECAG00000011226 |  |
| 20 | 48535577 | 48549772 | GLYATL3 | ENSECAG00000011755 |  |
| 20 | 48575803 | 48576378 | C20H6orf141 | ENSECAG00000011794 |  |
| 20 | 48613312 | 48613443 | U4 | ENSECAG00000026654 |  |
| 20 | 49089582 | 49094736 |  | ENSECAG00000042016 |  |
| 20 | 49597419 | 49618646 |  | ENSECAG00000032561 |  |
| 20 | 49630252 | 49685050 | TFAP2D | ENSECAG00000012285 |  |
| 20 | 49722940 | 49750398 | TFAP2B | ENSECAG00000015046 |  |
| 20 | 49762373 | 50058510 |  | ENSECAG00000031392 |  |
| 20 | 50225925 | 50255936 |  | ENSECAG00000027908 |  |
| 23 | 14012228 | 14252373 | PRUNE2 | ENSECAG00000010004 |  |
| 23 | 14448649 | 14454995 |  | ENSECAG00000037215 |  |
| 23 | 15021707 | 15131789 |  | ENSECAG00000031657 |  |
| 23 | 15524333 | 15544628 | NMRK1 | ENSECAG00000007336 |  |
| 23 | 15569707 | 15665310 | CARNMT1 | ENSECAG00000013964 |  |
| 23 | 15712916 | 15730526 | ANXA1 | ENSECAG00000015794 |  |
| 23 | 15807696 | 15810845 |  | ENSECAG00000040848 |  |
| 23 | 15816273 | 15818877 |  | ENSECAG00000041280 |  |
| 23 | 15836725 | 15846388 |  | ENSECAG00000029009 |  |
| 23 | 15915068 | 15987845 |  | ENSECAG00000036411 |  |
| 23 | 16153995 | 16295172 |  | ENSECAG00000036718 |  |
| 23 | 16849076 | 16892439 |  | ENSECAG00000029858 |  |
| 23 | 16921205 | 17098343 | RORB | ENSECAG00000024312 |  |
| 23 | 17367547 | 17369991 |  | ENSECAG00000038328 |  |
| 23 | 17494268 | 17625036 | ALDH1A1 | ENSECAG00000019343 |  |
| 23 | 17908434 | 17943432 |  | ENSECAG00000043198 |  |
| 23 | 18064062 | 18074544 | ZFAND5 | ENSECAG00000023826 |  |
| 23 | 18468208 | 18512201 | ABHD17B | ENSECAG00000020790 |  |
| 23 | 18491976 | 18492108 | eca-mir-8951 | ENSECAG00000029458 |  |
| 23 | 18615027 | 18670051 | CEMIP2 | ENSECAG00000021484 |  |
| 23 | 18881941 | 19728032 | TRPM3 | ENSECAG00000008135 |  |
| 23 | 19459692 | 19459801 |  | ENSECAG00000030779 |  |
| 23 | 19484076 | 19489325 |  | ENSECAG00000037389 |  |
| 23 | 19521519 | 19521628 |  | ENSECAG00000028185 |  |
| 23 | 19824603 | 19846076 | KLF9 | ENSECAG00000024925 |  |
| 23 | 20364092 | 20405123 | PTAR1 | ENSECAG00000021853 |  |
| 23 | 20561708 | 20652475 | APBA1 | ENSECAG00000000271 |  |
| 23 | 20671595 | 20675476 |  | ENSECAG00000032124 |  |
| 23 | 21436265 | 21439244 |  | ENSECAG00000033966 |  |
| 23 | 21444589 | 21452537 |  | ENSECAG00000033647 |  |
| 23 | 21474038 | 21478432 | TMEM252 | ENSECAG00000010049 |  |
| 23 | 21687642 | 21689465 |  | ENSECAG00000037327 |  |
| 23 | 21769101 | 21983585 | DOCK8 | ENSECAG00000011917 |  |
| 23 | 22016583 | 22017689 |  | ENSECAG00000040047 |  |
| 23 | 22044313 | 22213482 | KANK1 | ENSECAG00000016219 |  |
| 23 | 22268427 | 22392510 | DMRT3 | ENSECAG00000023412 |  |
| 23 | 22410347 | 22420690 |  | ENSECAG00000038701 |  |
| 23 | 22447169 | 22454628 | DMRT2 | ENSECAG00000035701 |  |
| 23 | 22641562 | 22798758 |  | ENSECAG00000040629 |  |
| 23 | 23220315 | 23226785 |  | ENSECAG00000038442 |  |
| 23 | 23273096 | 23425258 | SMARCA2 | ENSECAG00000024187 |  |
| 23 | 23784757 | 23813956 | VLDLR | ENSECAG00000021859 |  |
| 23 | 23869786 | 23881506 | KCNV2 | ENSECAG00000016602 |  |
| 23 | 24704592 | 24706818 |  | ENSECAG00000040492 |  |
| 23 | 25410619 | 25519998 | SLC1A1 | ENSECAG00000010935 |  |
| 23 | 25579181 | 25580065 | PLPP6 | ENSECAG00000023969 |  |
| 23 | 25594600 | 25612572 | CDC37L1 | ENSECAG00000000269 |  |
| 23 | 25669428 | 25738214 | RCL1 | ENSECAG00000015973 |  |
| 23 | 25728381 | 25728471 | MIR101-2 | ENSECAG00000025269 |  |
| 23 | 25864367 | 25998464 | JAK2 | ENSECAG00000009119 |  |
| 23 | 26165729 | 26183867 | CD274 | ENSECAG00000016312 |  |
| 23 | 26234918 | 26281981 | PDCD1LG2 | ENSECAG00000020578 |  |
| 23 | 26292004 | 26292110 | U6 | ENSECAG00000039561 |  |
| 23 | 26322452 | 26324218 |  | ENSECAG00000032275 |  |
| 23 | 26324239 | 26501791 | RIC1 | ENSECAG00000006558 |  |
| 23 | 26594216 | 26596228 |  | ENSECAG00000028502 |  |
| 23 | 26609104 | 26619679 | MLANA | ENSECAG00000009103 |  |
| 23 | 26871150 | 26882567 | IL33 | ENSECAG00000010475 |  |
| 23 | 26915595 | 26920992 |  | ENSECAG00000030678 |  |
| 23 | 27022442 | 27025624 |  | ENSECAG00000042096 |  |
| 23 | 27055309 | 27173813 | UHRF2 | ENSECAG00000013985 |  |
| 23 | 27368361 | 27722943 | KDM4C | ENSECAG00000024063 |  |
| 23 | 27864566 | 27900196 |  | ENSECAG00000028250 |  |
| 23 | 27995465 | 28001286 |  | ENSECAG00000042943 |  |
| 23 | 28204007 | 28463435 |  | ENSECAG00000043568 |  |
| 23 | 28301777 | 28534718 |  | ENSECAG00000039711 |  |
| 23 | 28307385 | 28494201 |  | ENSECAG00000033599 |  |
| 23 | 28686266 | 28720062 |  | ENSECAG00000034453 |  |
| 23 | 29250525 | 29262919 |  | ENSECAG00000041274 |  |
| 23 | 31304828 | 31313098 |  | ENSECAG00000028066 |  |
| 23 | 31440789 | 31497801 |  | ENSECAG00000038514 |  |
| 23 | 32594790 | 32608964 |  | ENSECAG00000035403 |  |
| 23 | 32618595 | 32636270 | TYRP1 | ENSECAG00000009440 |  |
| 23 | 32689722 | 32737256 | LURAP1L | ENSECAG00000008425 |  |
| 23 | 33146959 | 33280889 |  | ENSECAG00000030863 |  |
| 23 | 33198728 | 33217640 |  | ENSECAG00000041584 |  |
| 23 | 34813960 | 34817967 |  | ENSECAG00000033807 |  |
