# Supplementary figures and images for: Runs of homozygosity in Sable Island feral horses reveal the genomic consequences of inbreeding and divergence from domestic breeds
Source: BMC Genomics. 2022 Jul 12;23:501. doi: 10.1186/s12864-022-08729-9 (PMC9275264; doi:10.1186/s12864-022-08729-9)

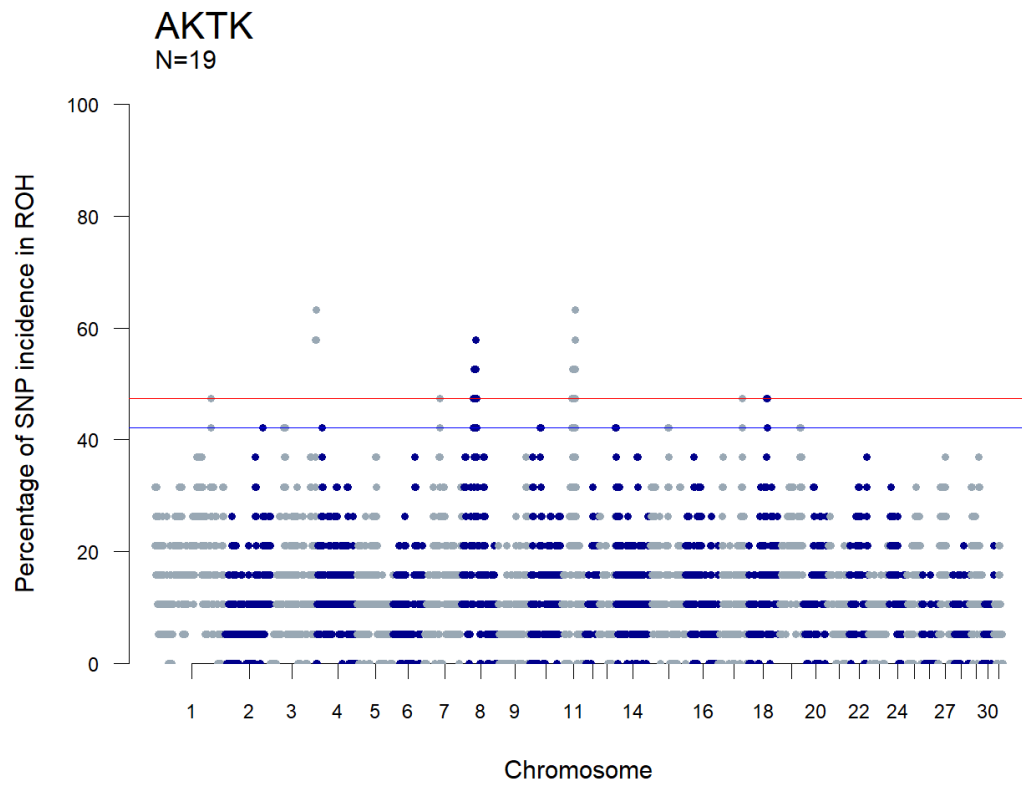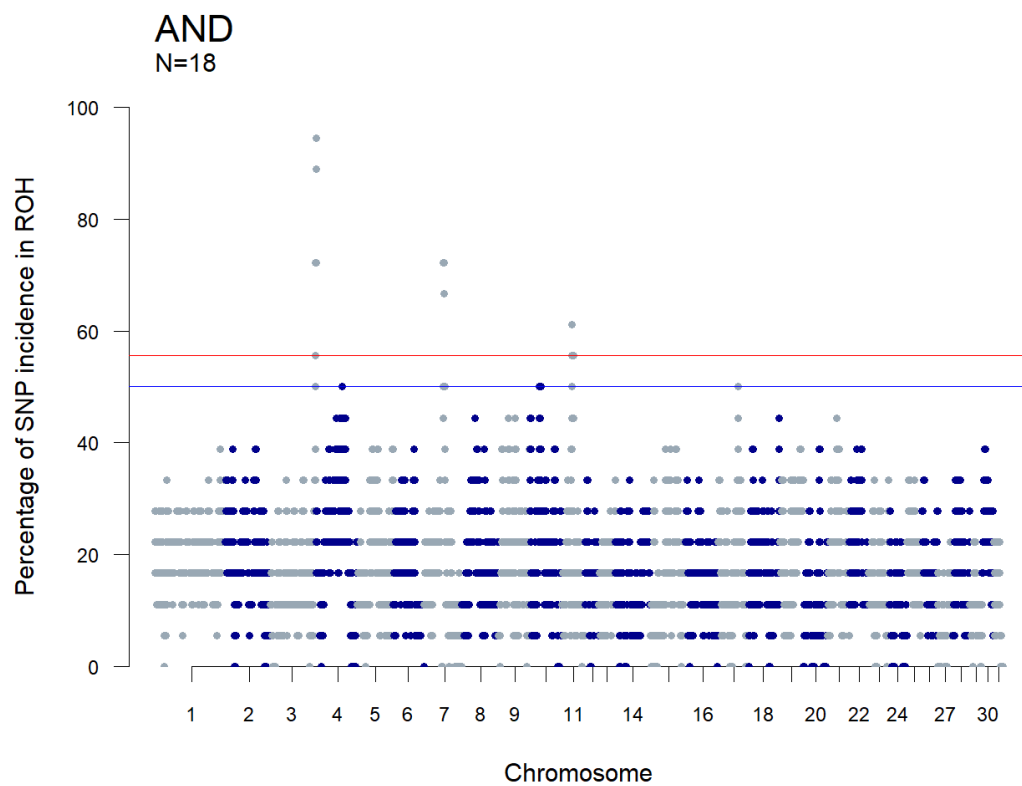

ARR  
N=24

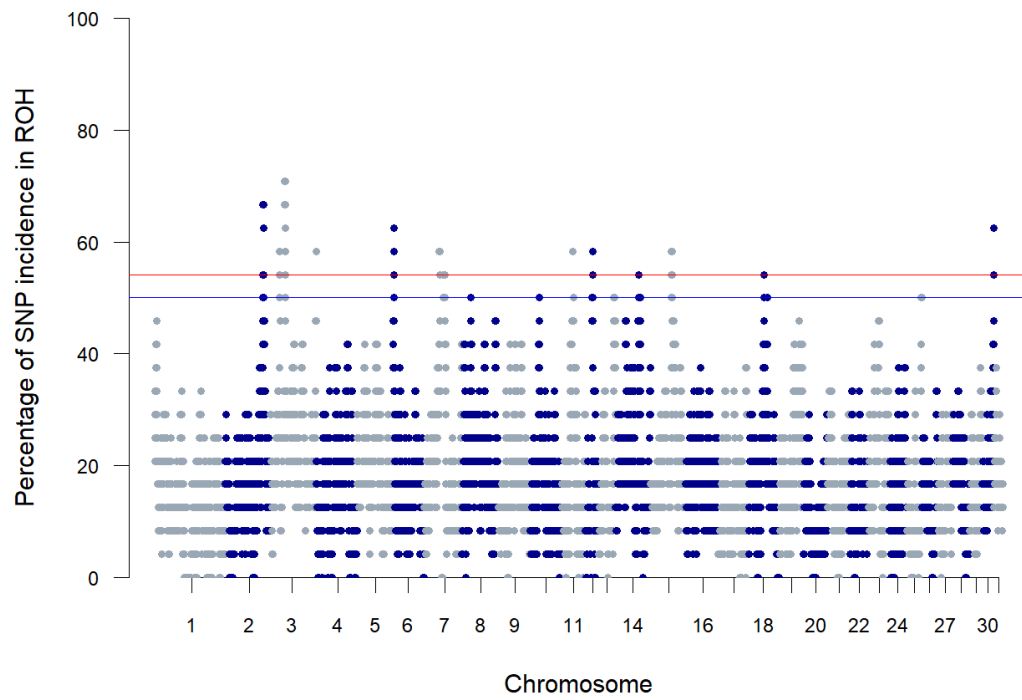

BEL  
N=30

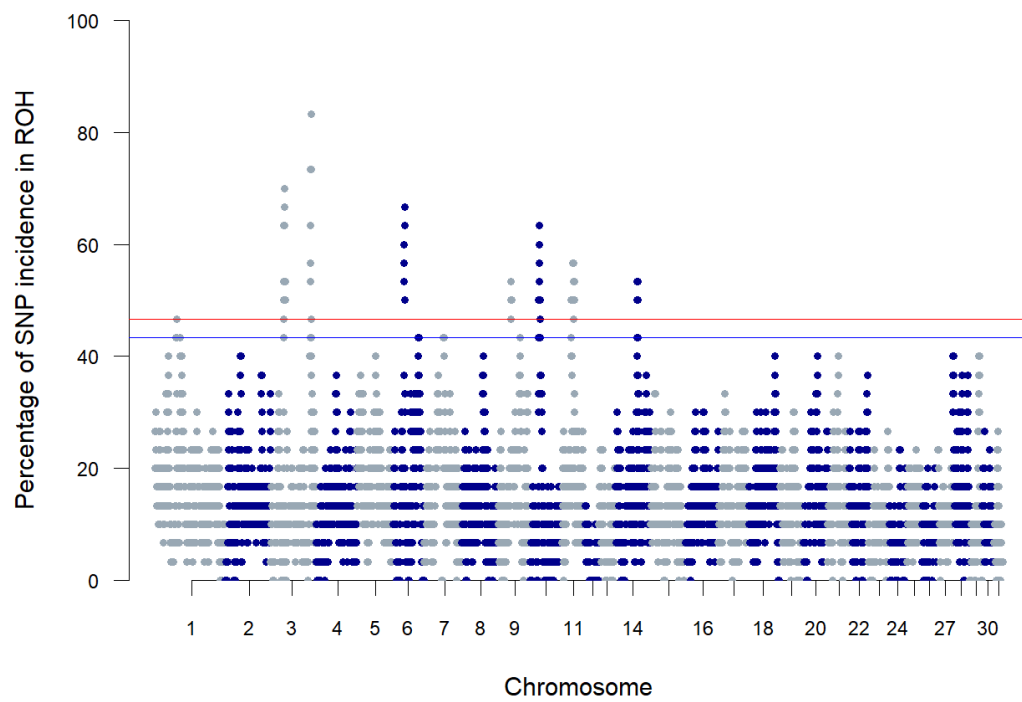

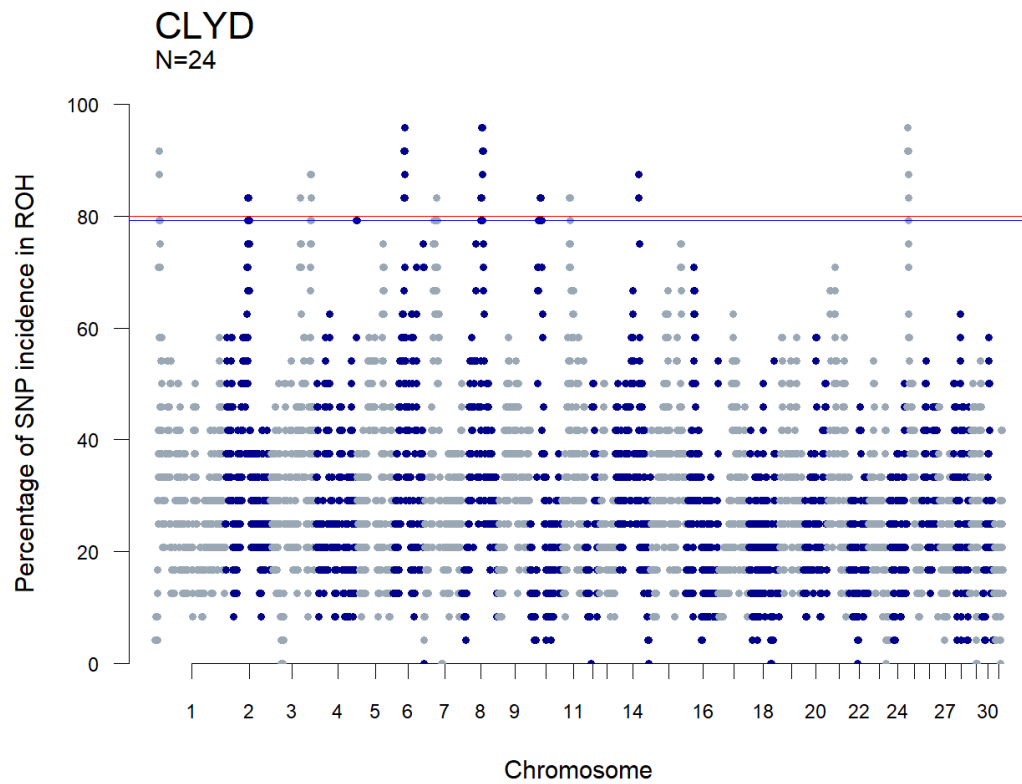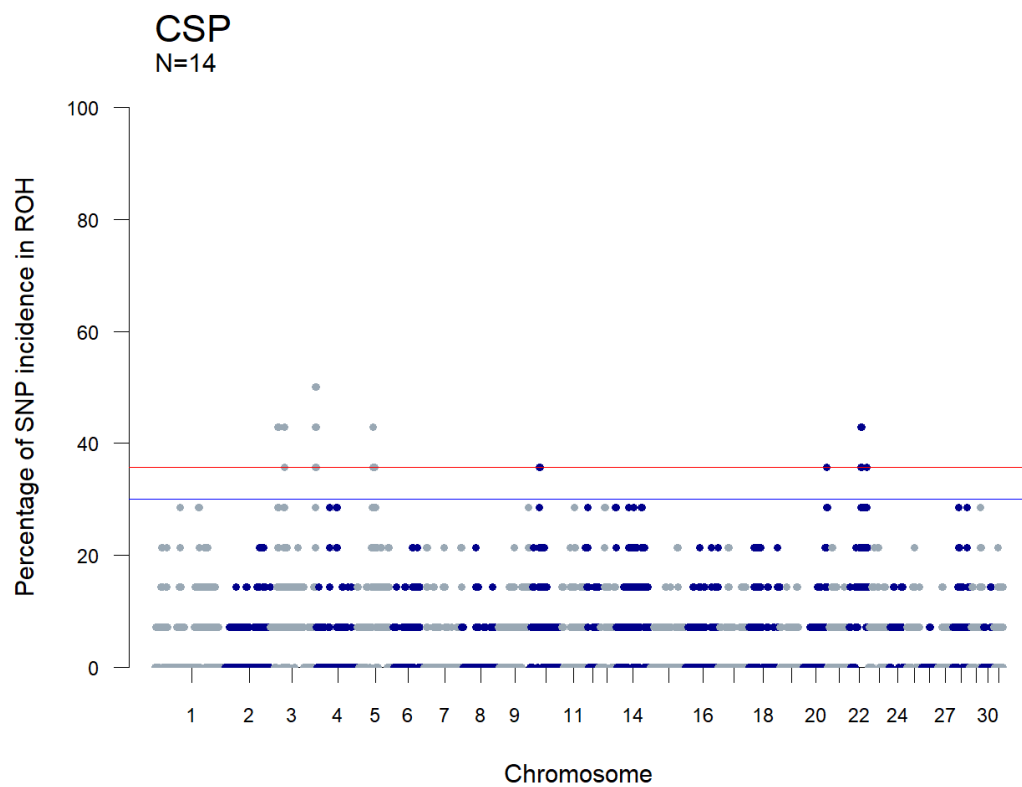

## EXMR N=24

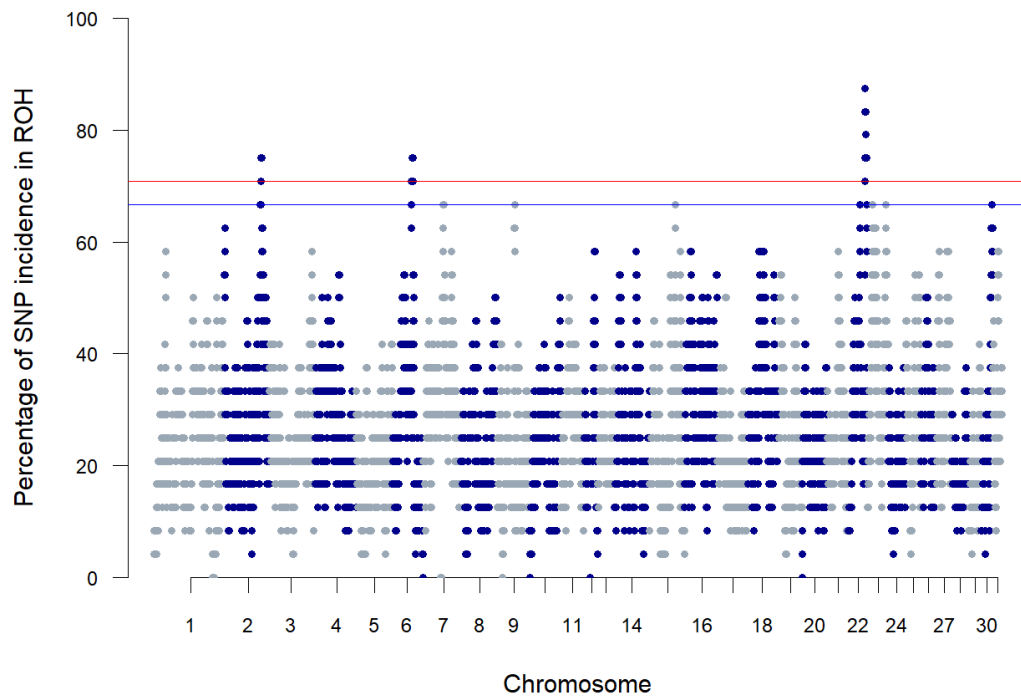

## FELL N=21

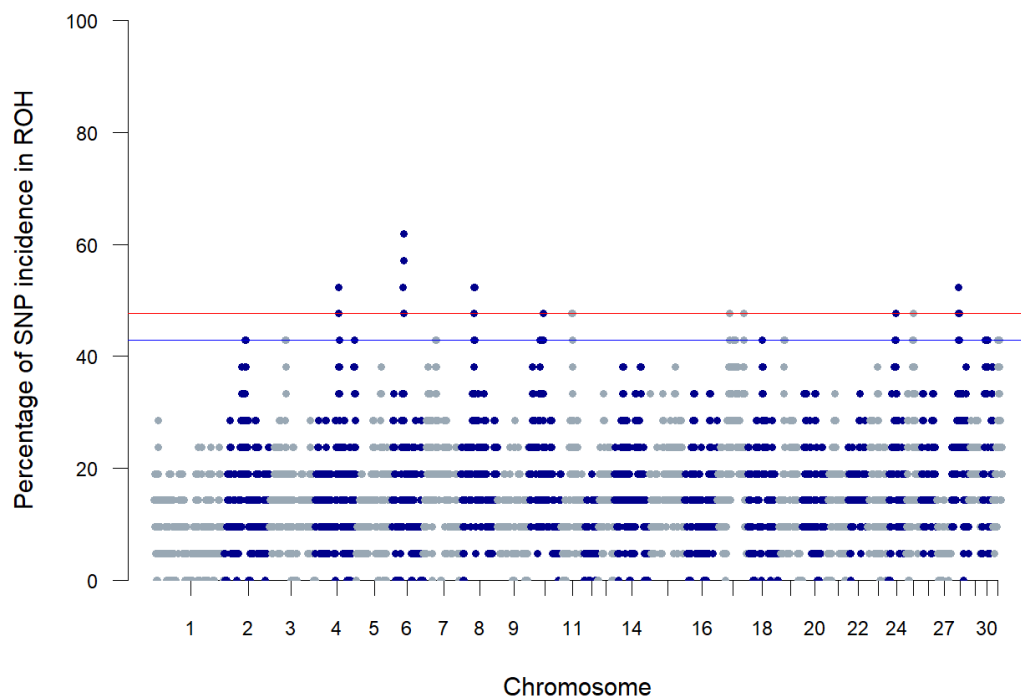

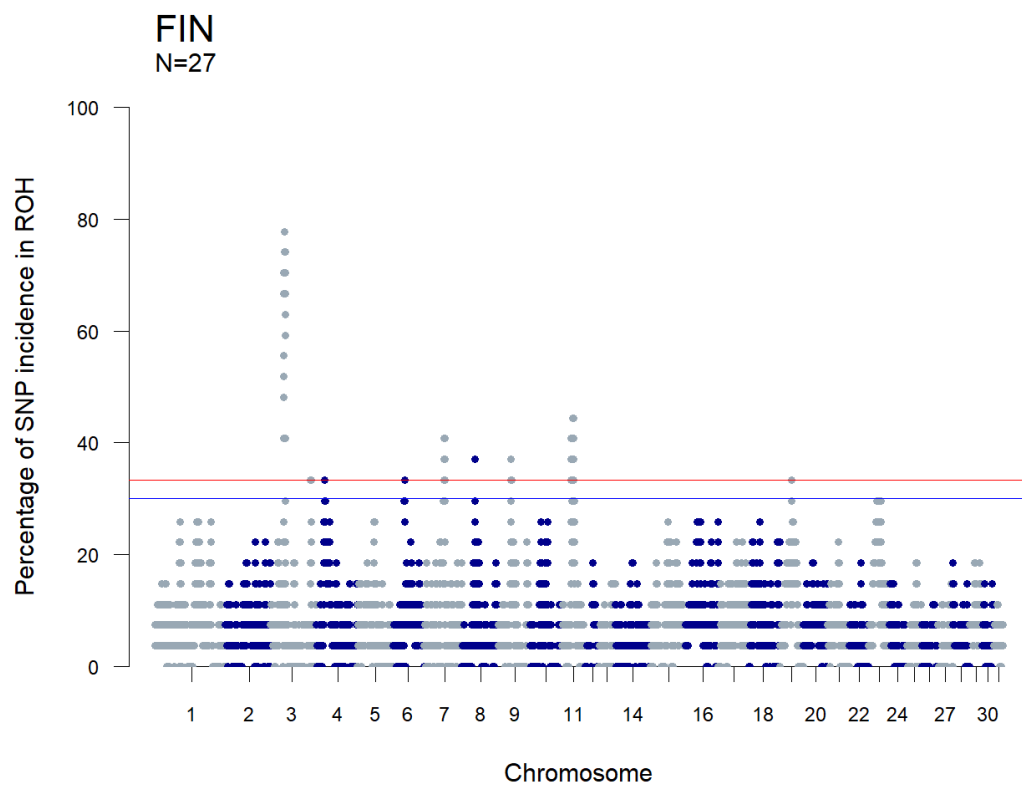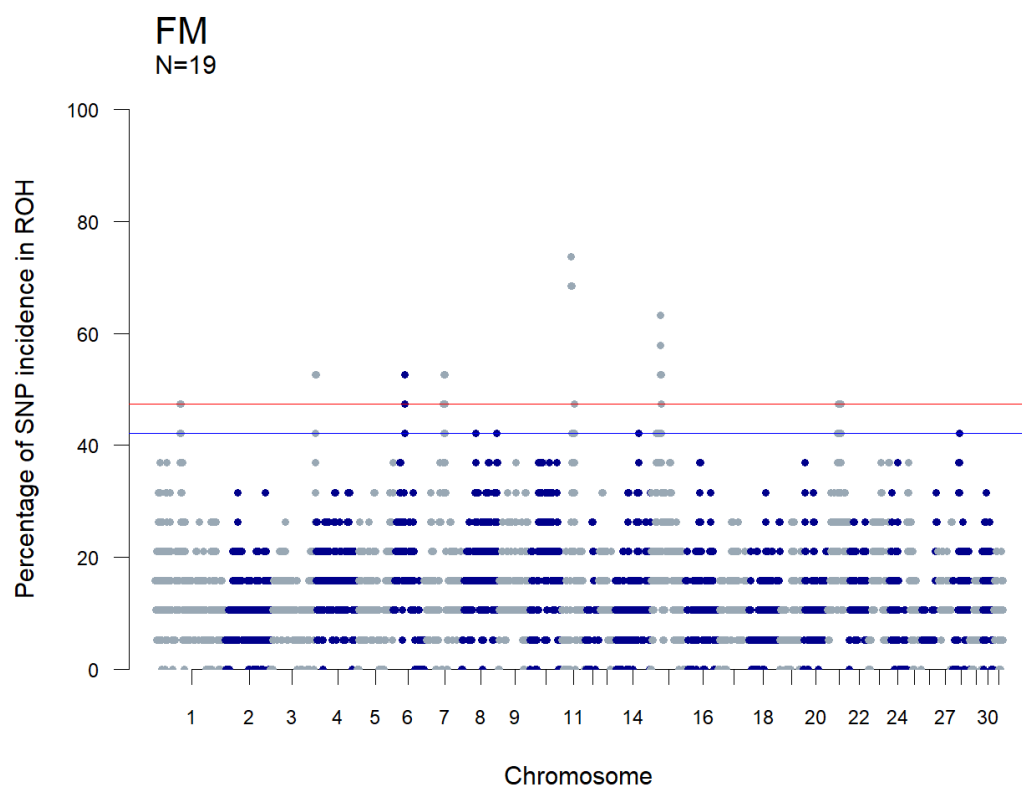

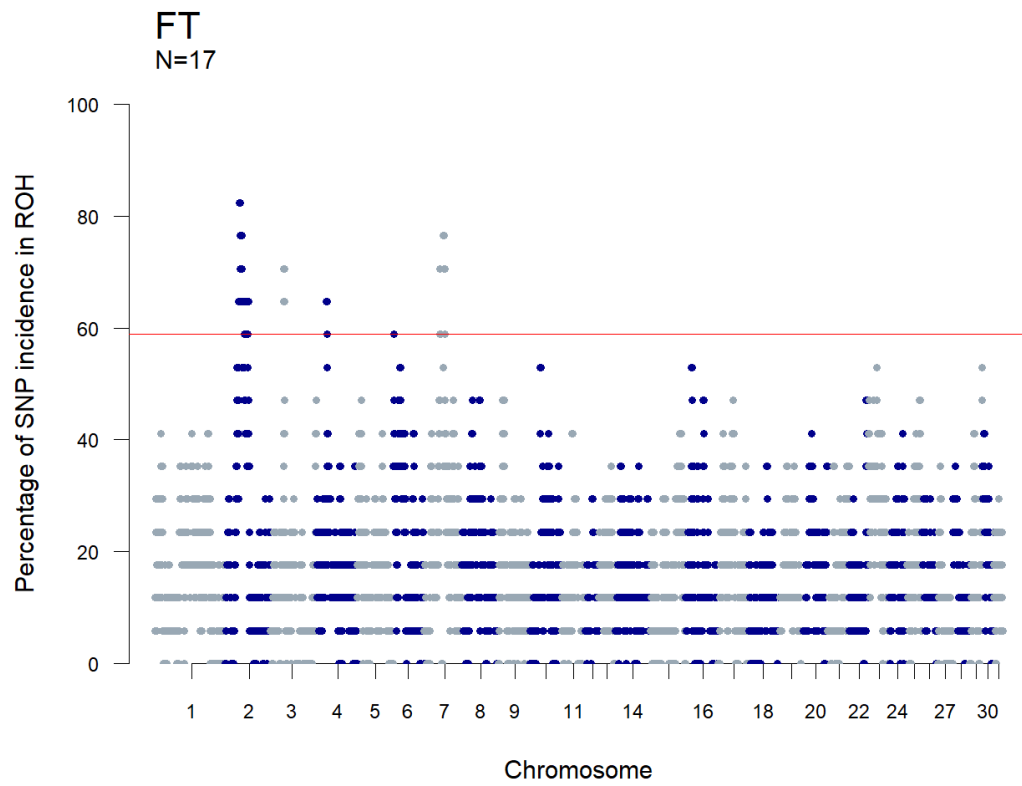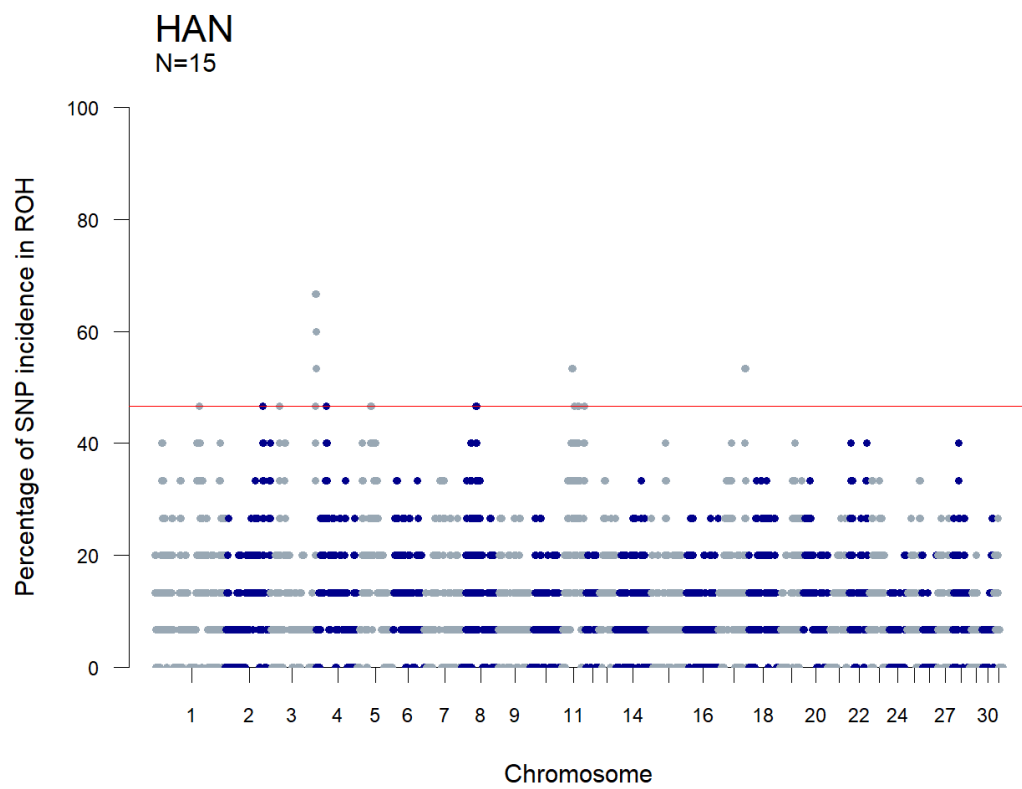

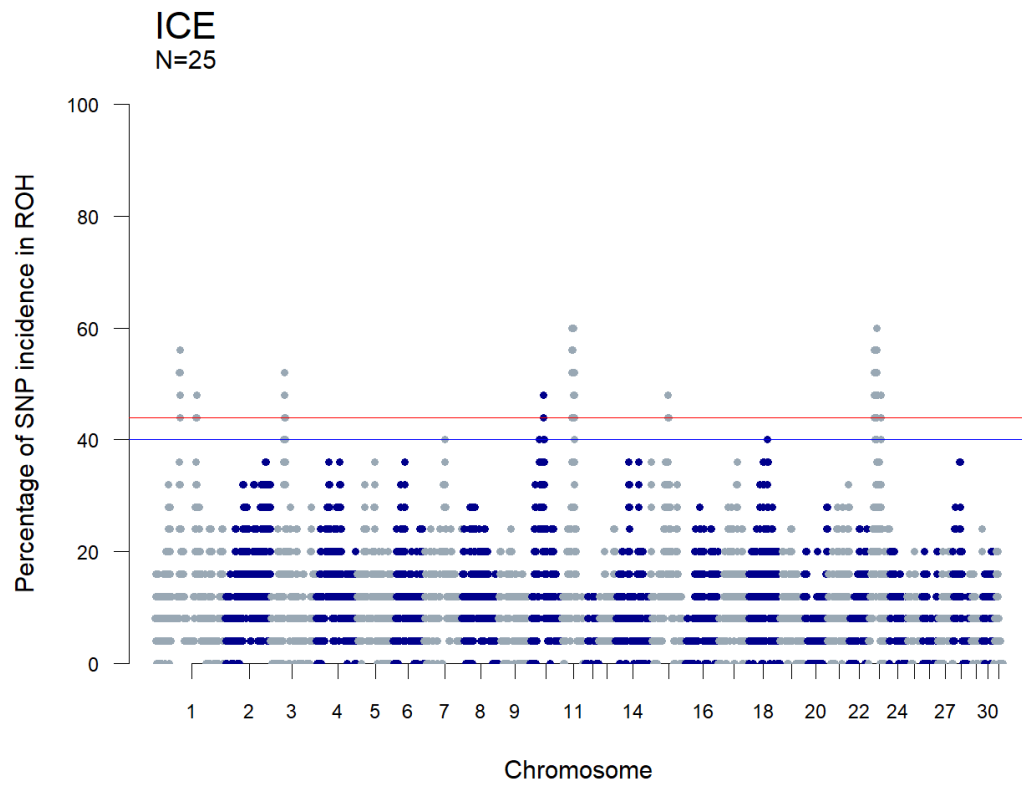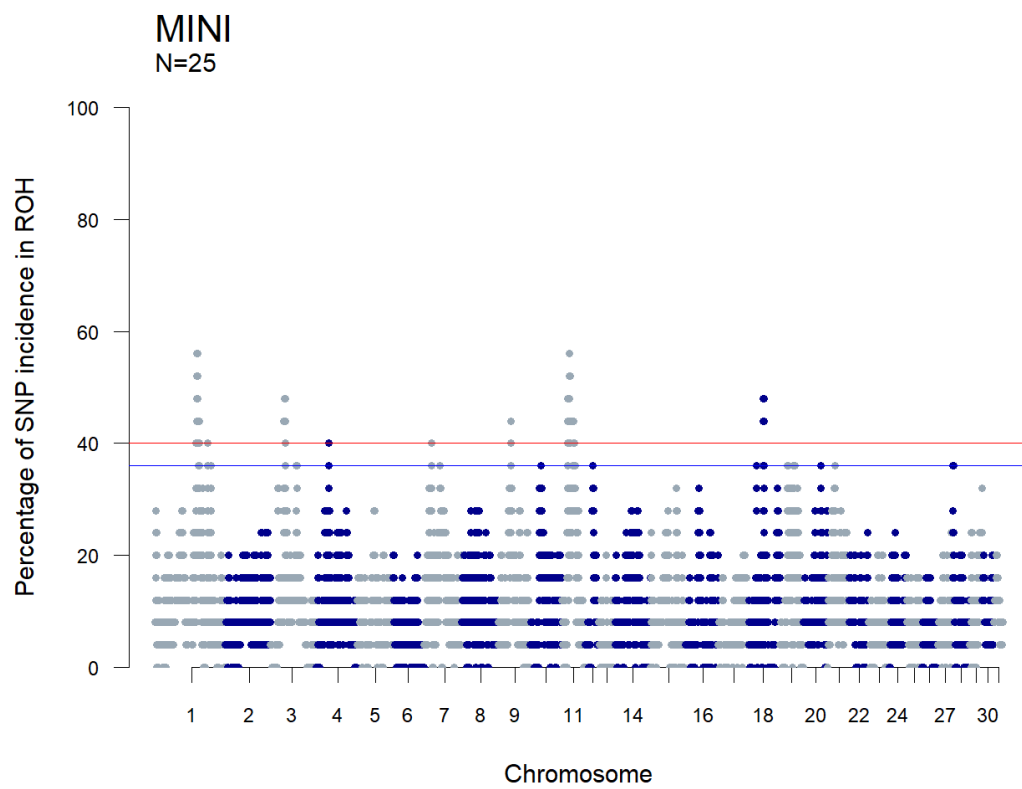

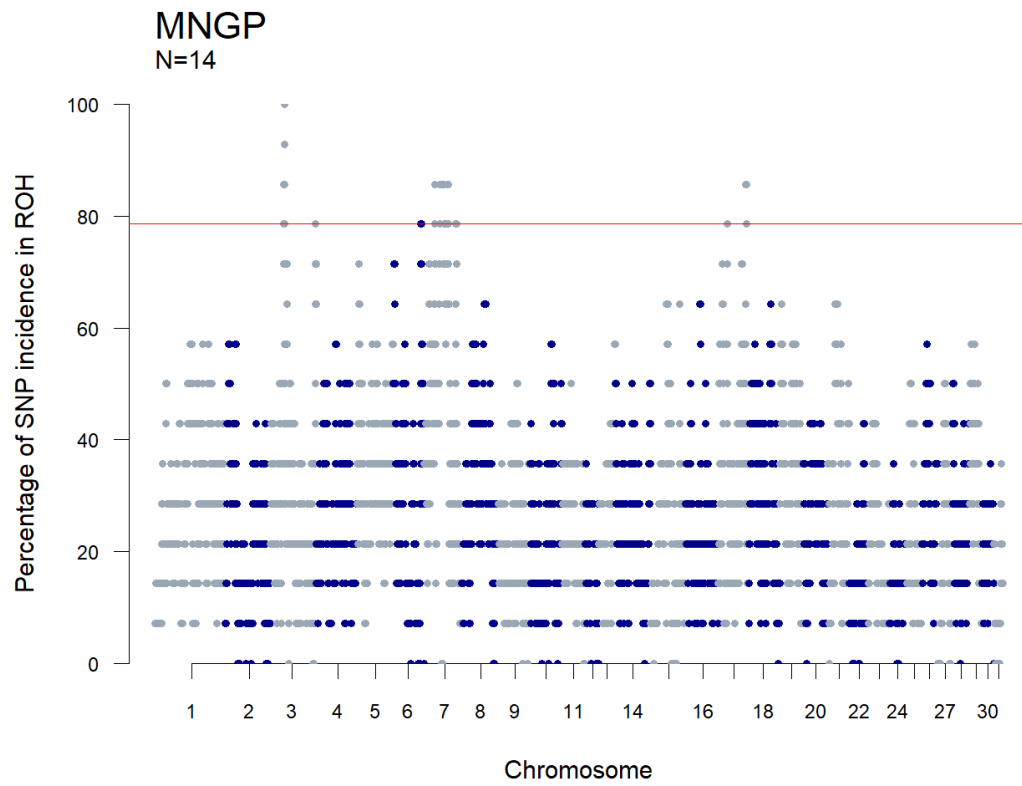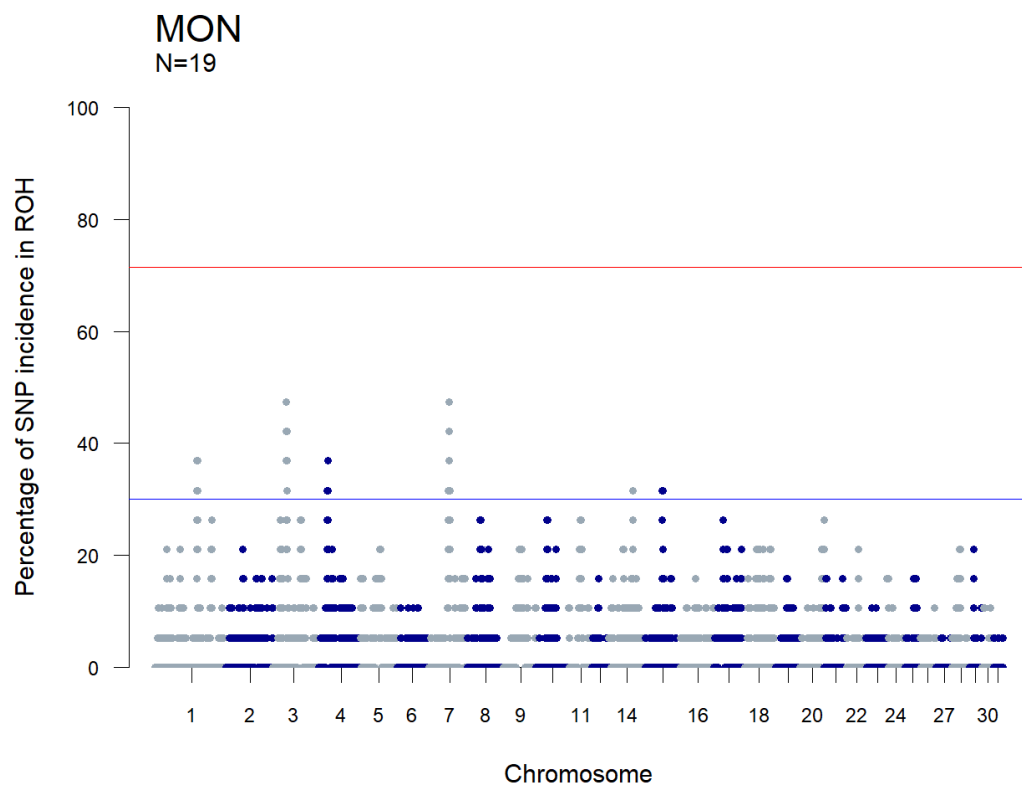

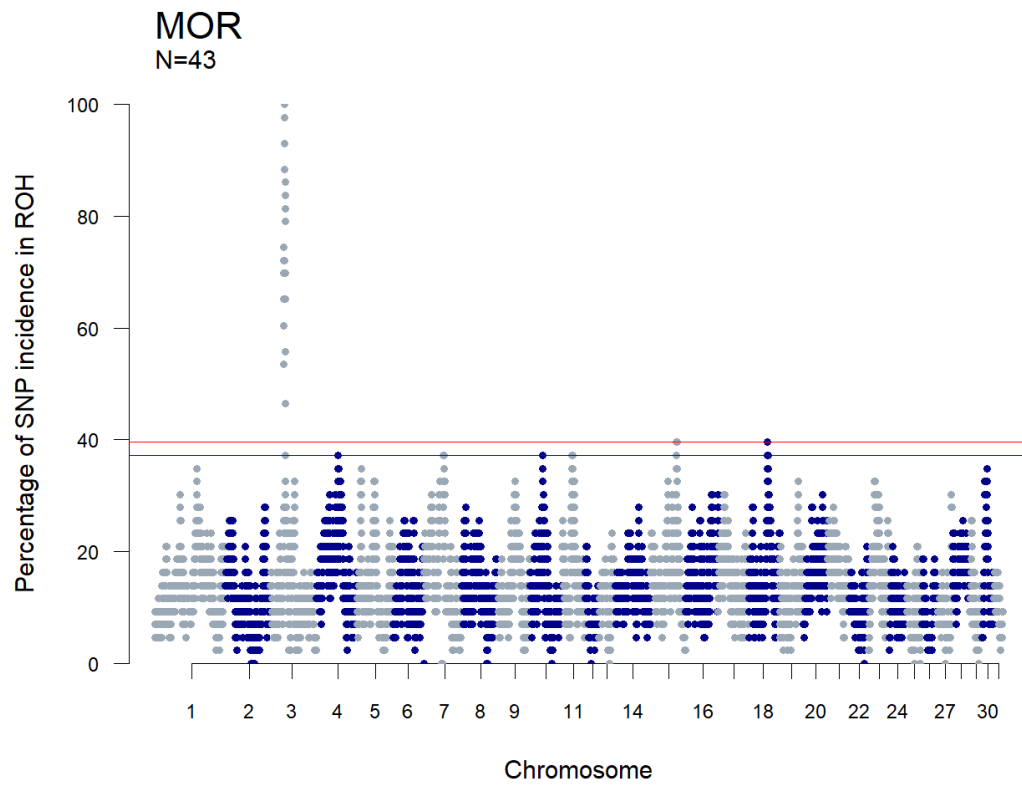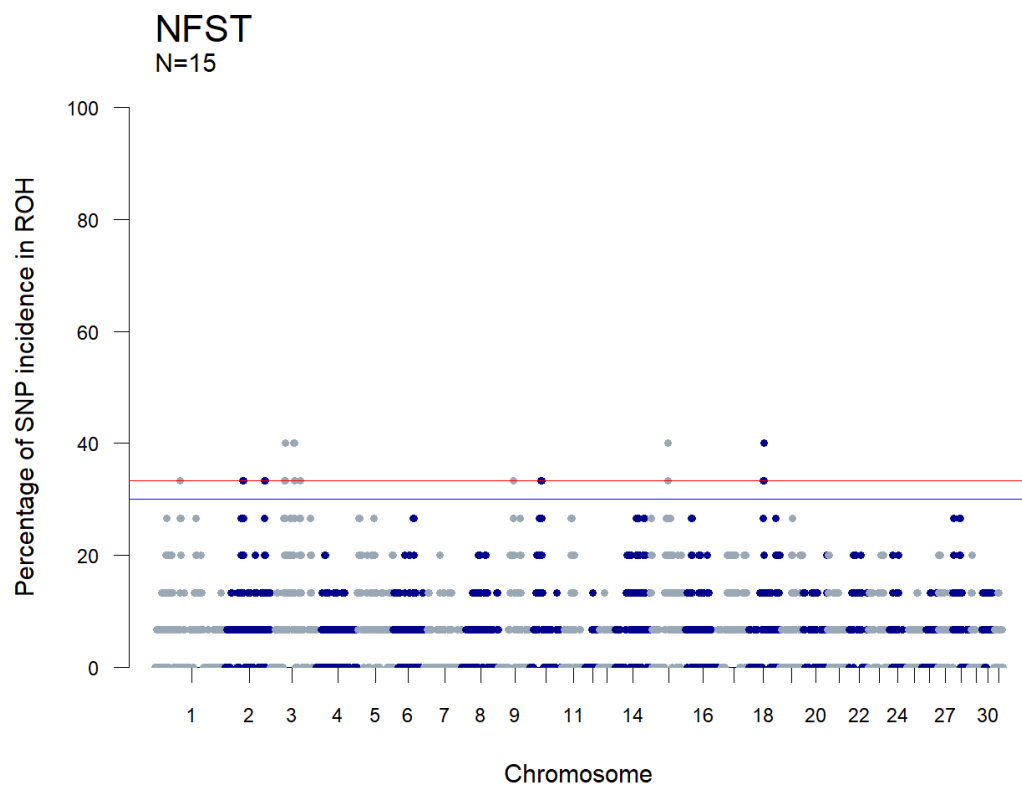

## NORF

N=21

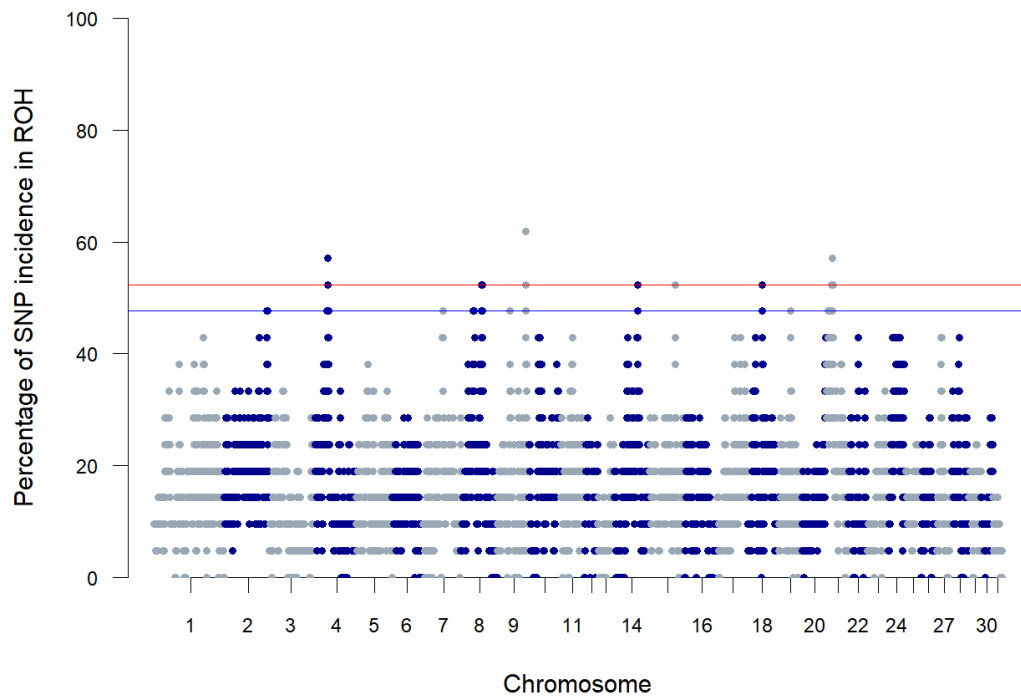

## NSWE

N=19

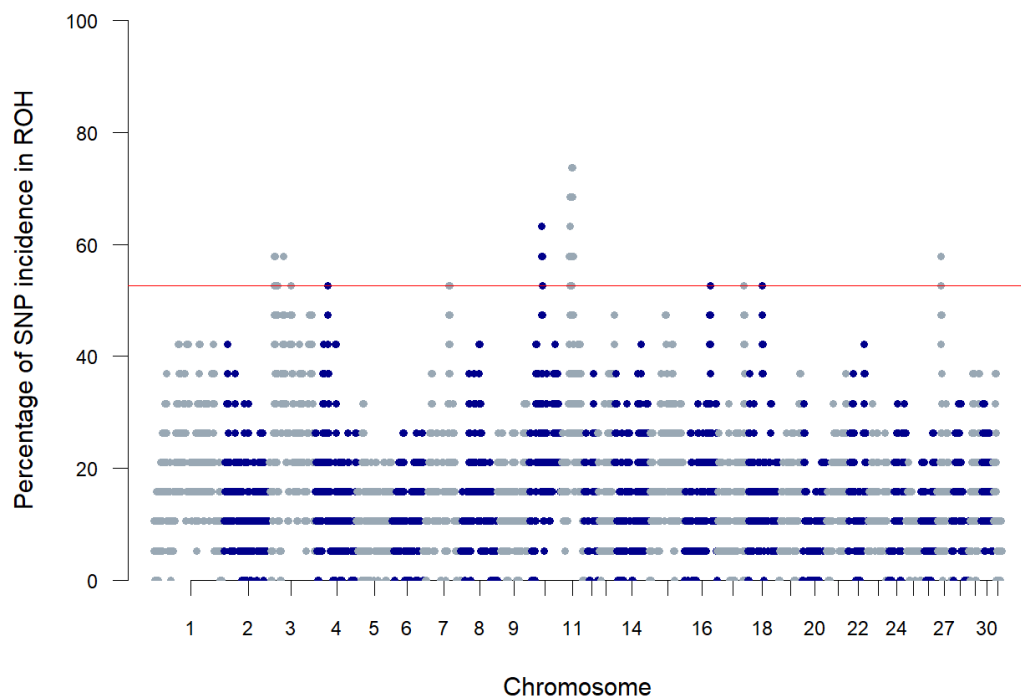

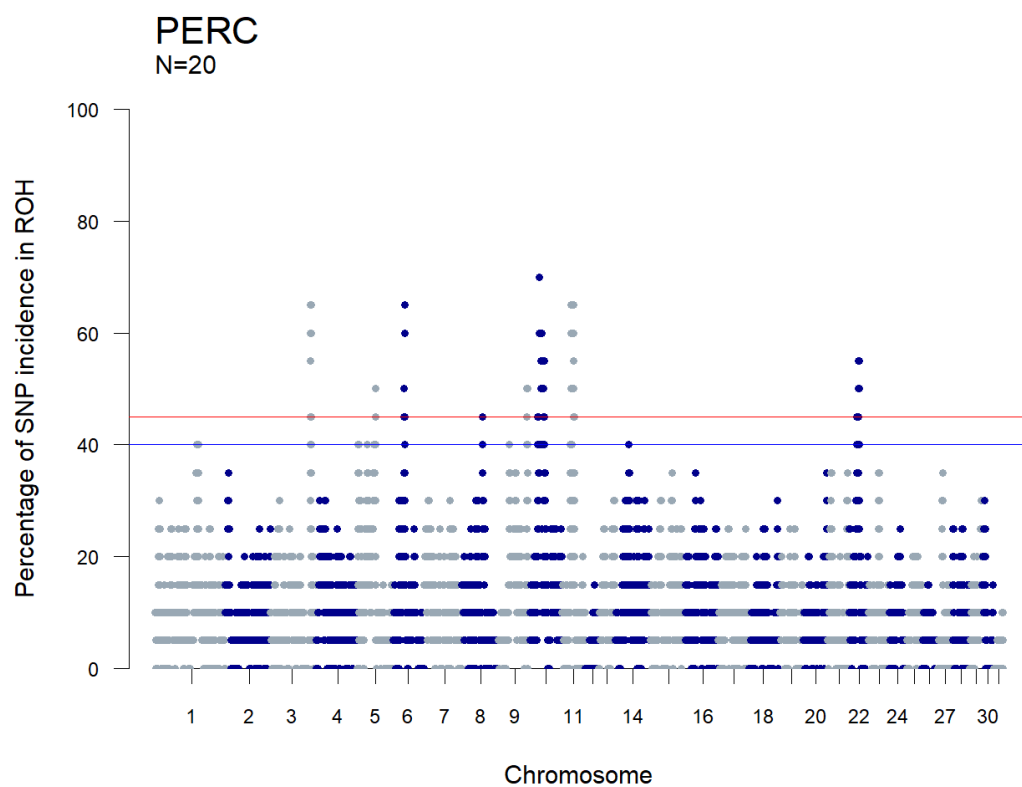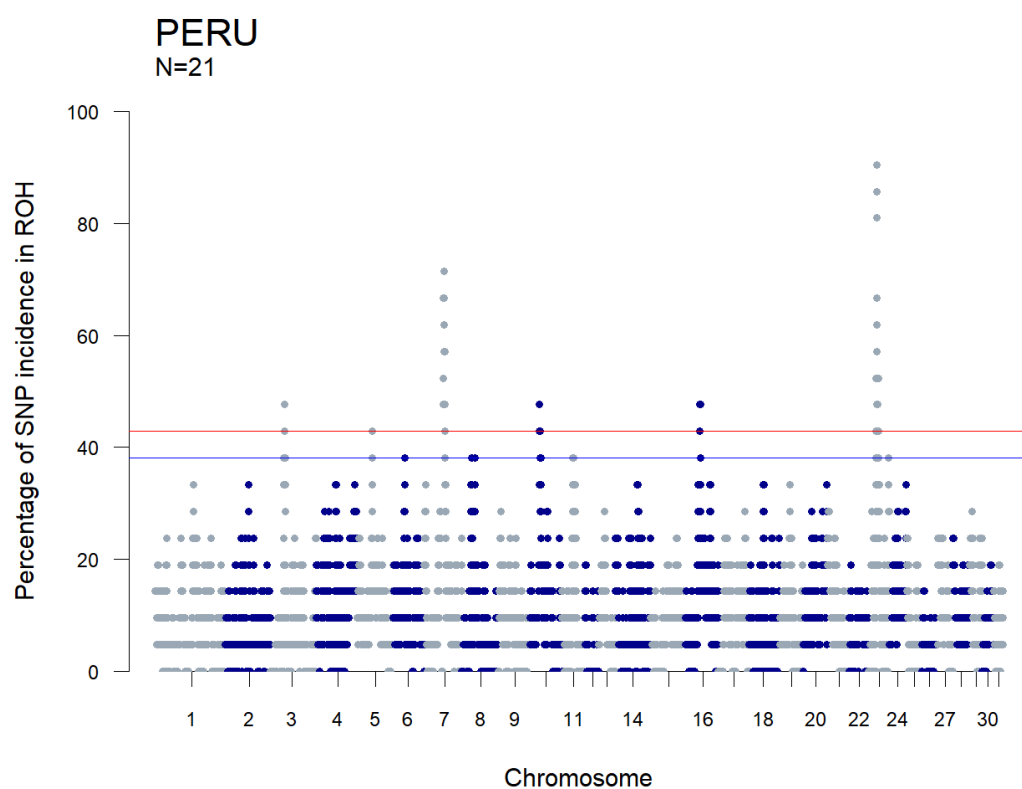

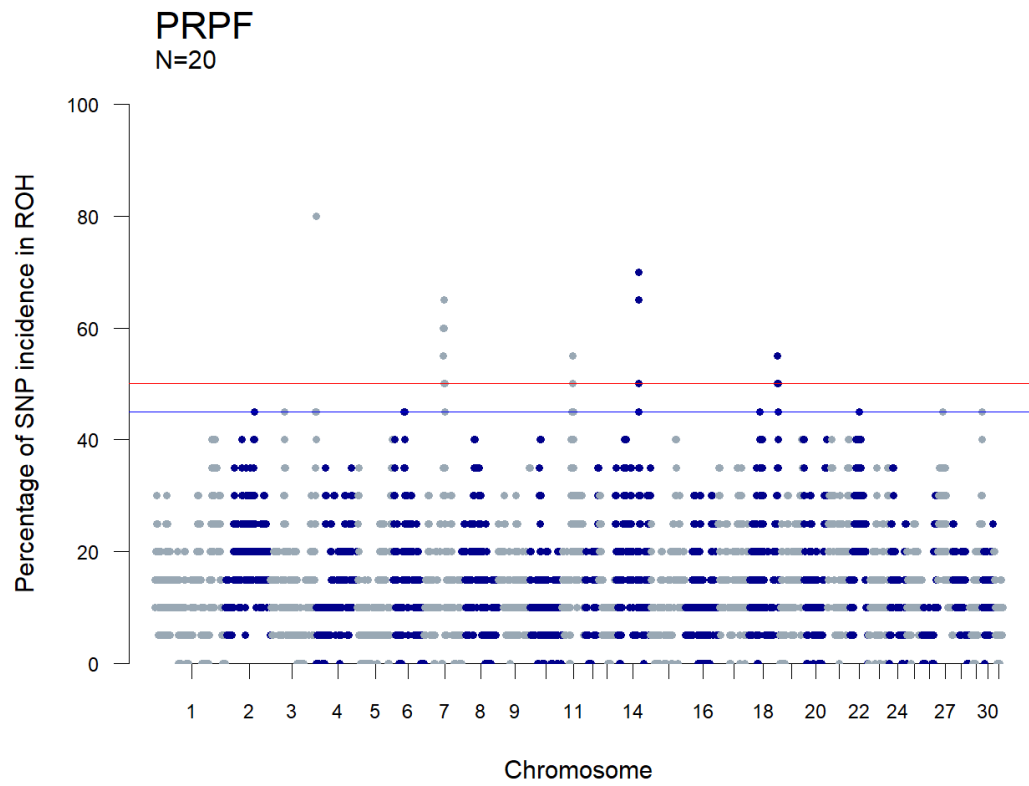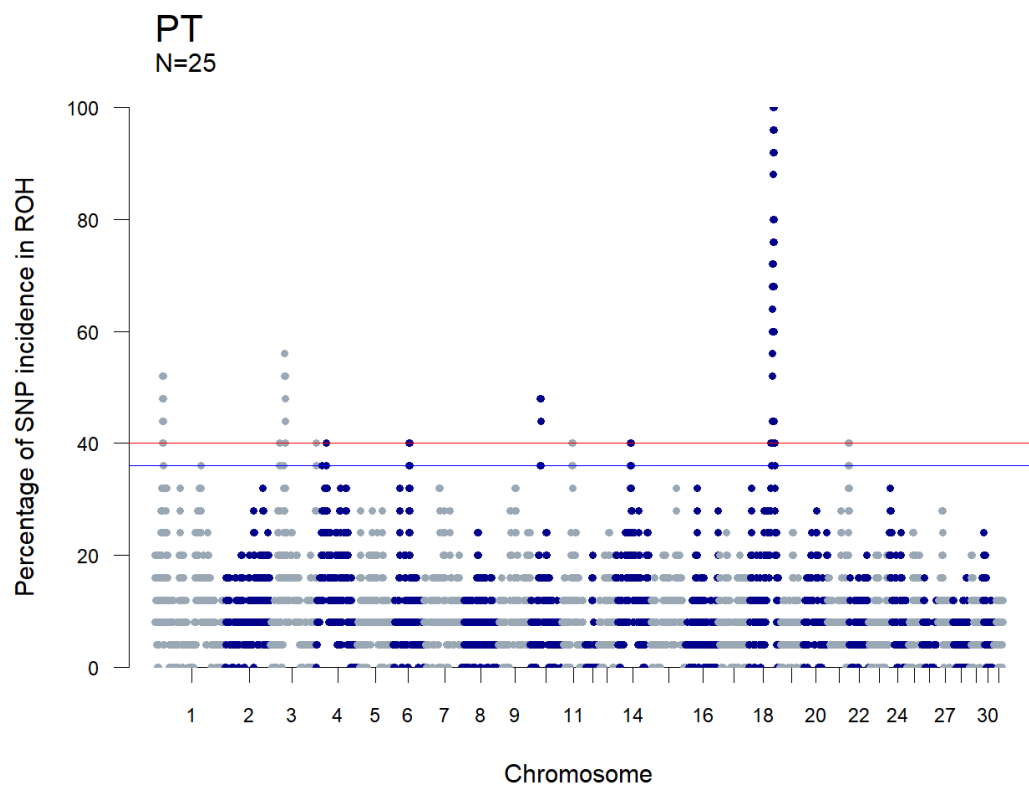

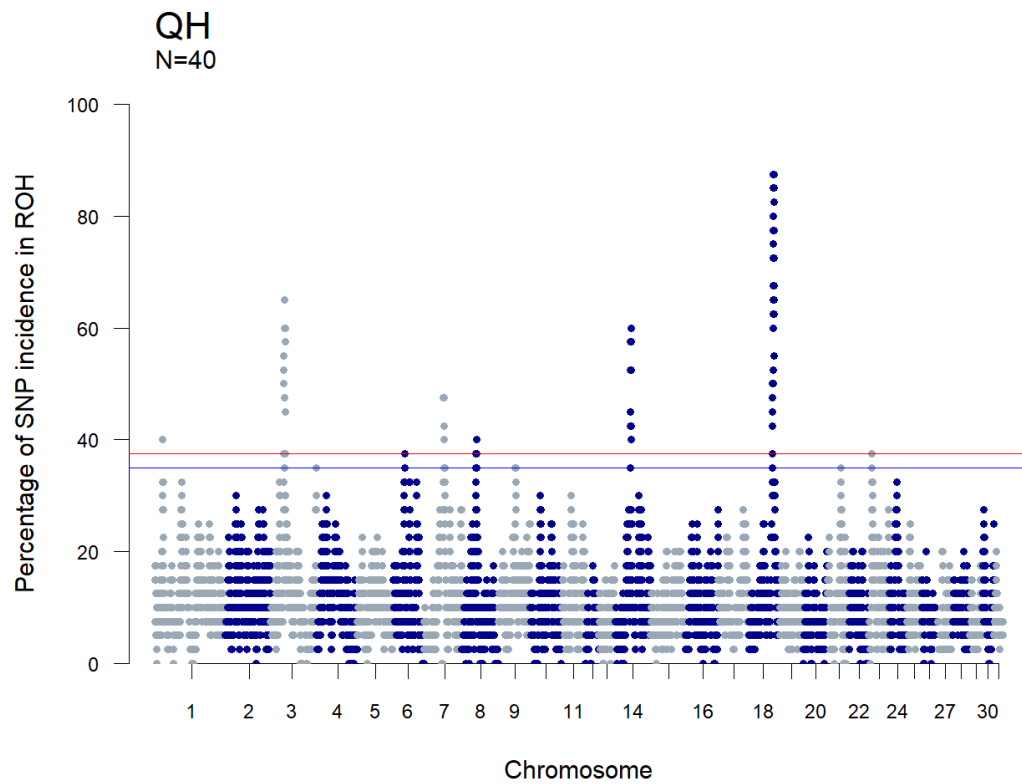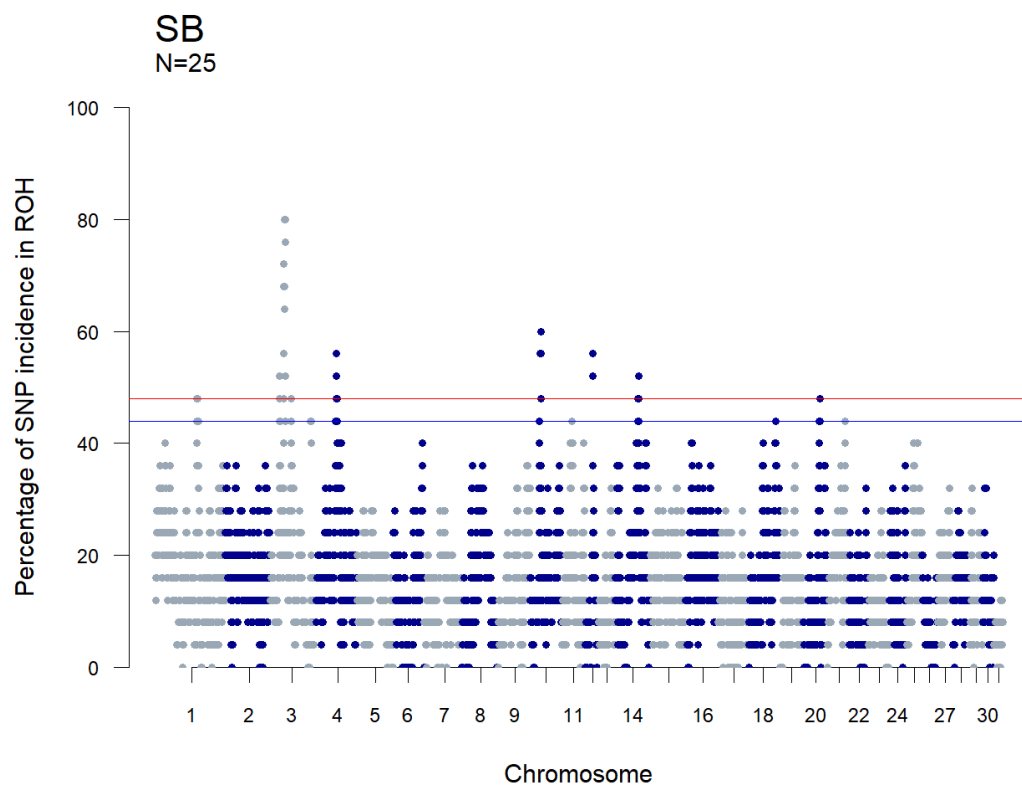

SHET  
N=27

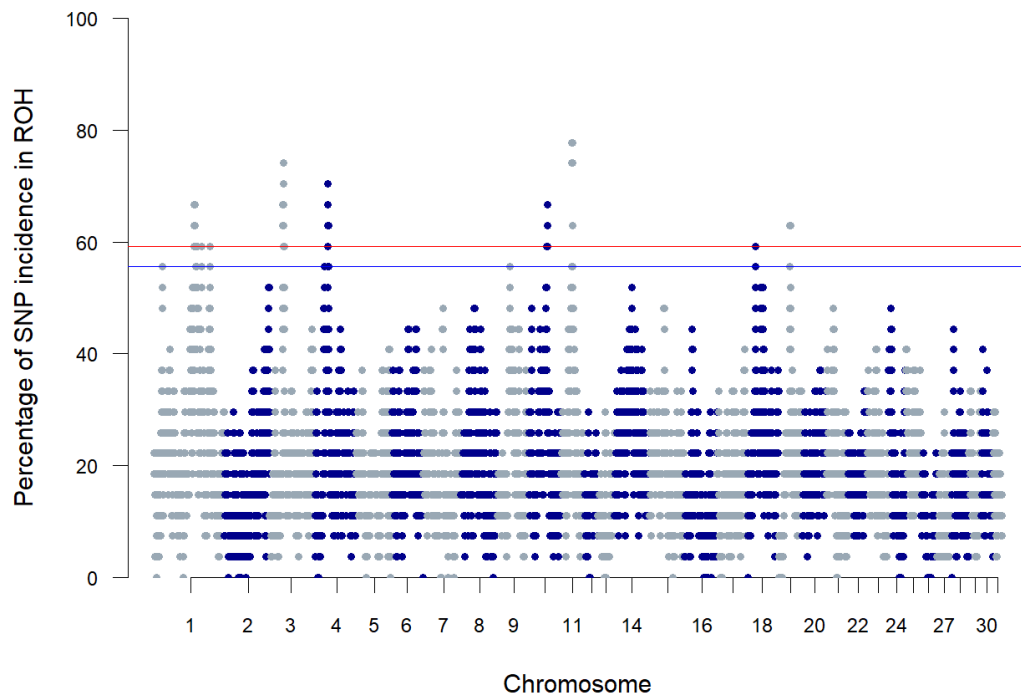

SHR  
N=22

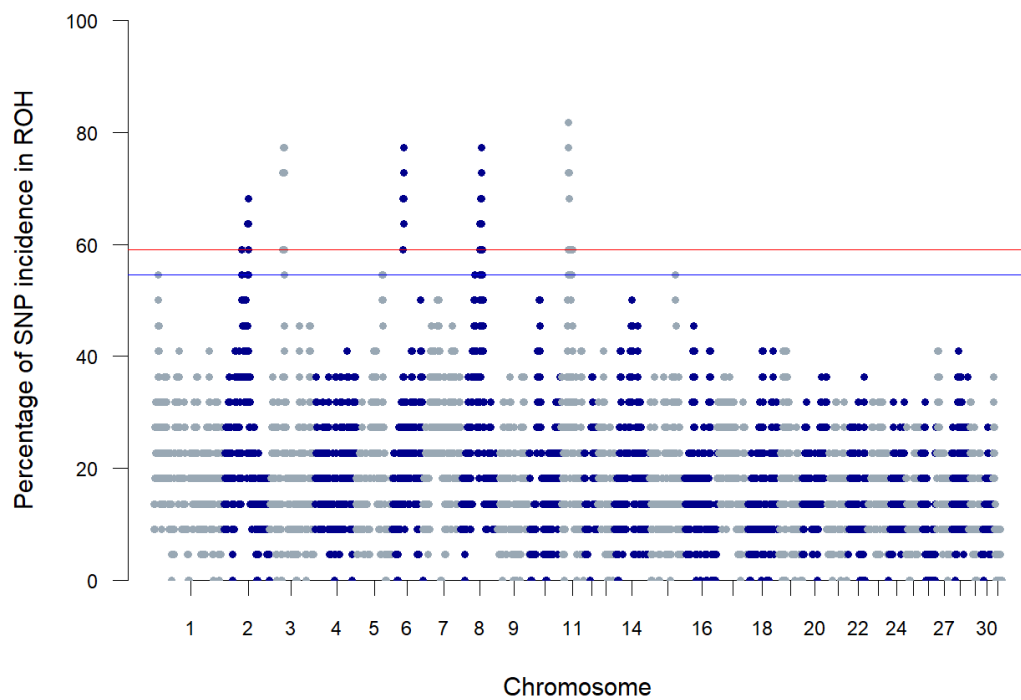

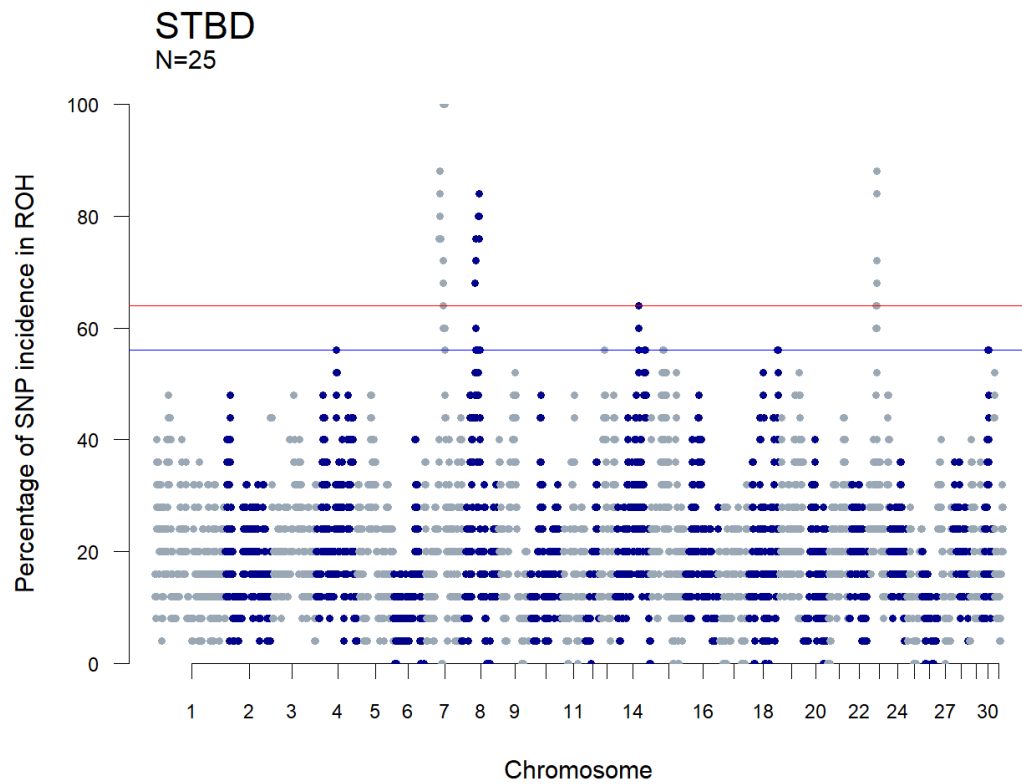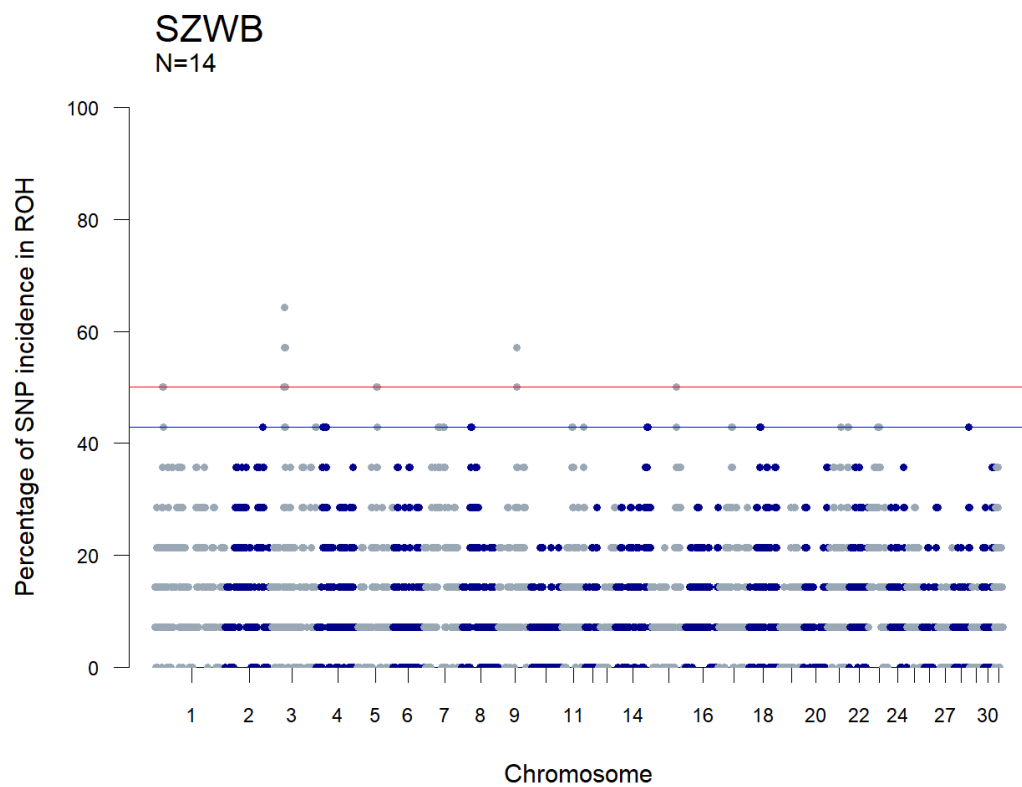

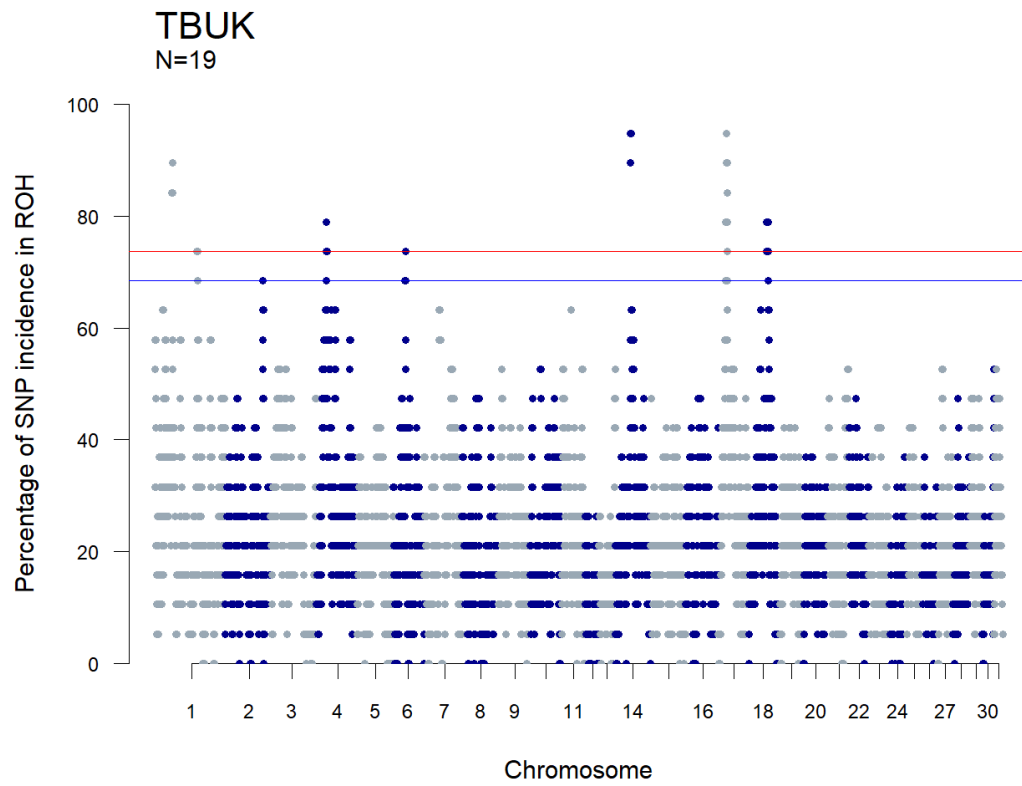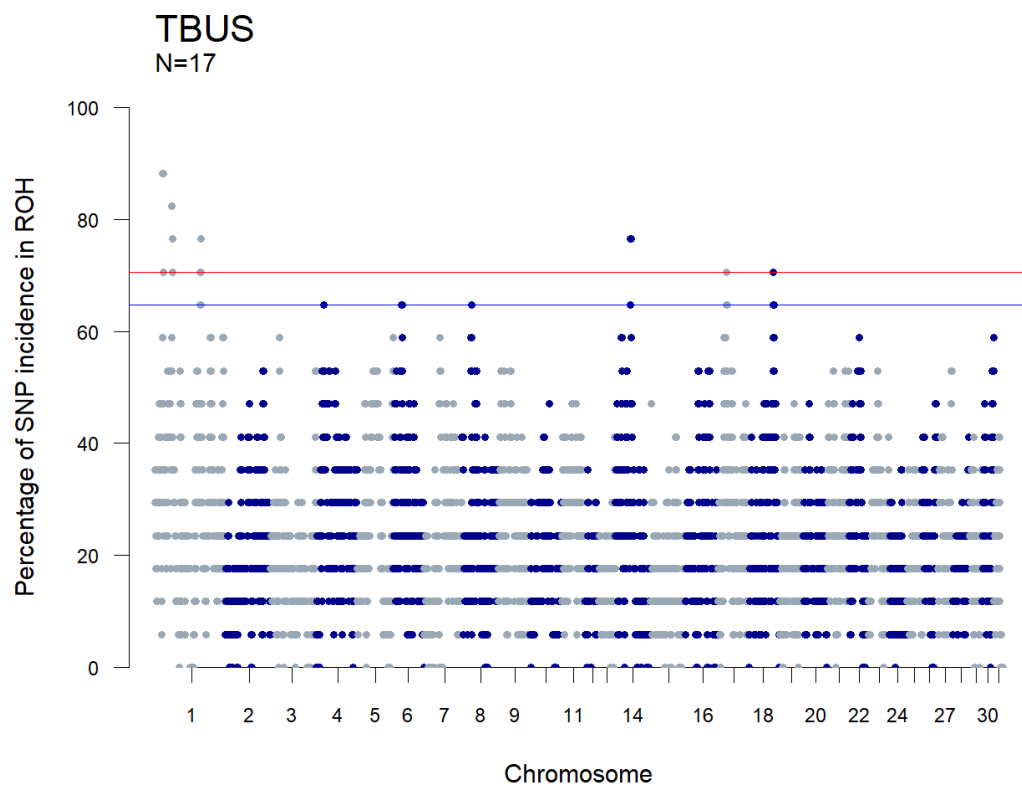

TUVA  
N=15

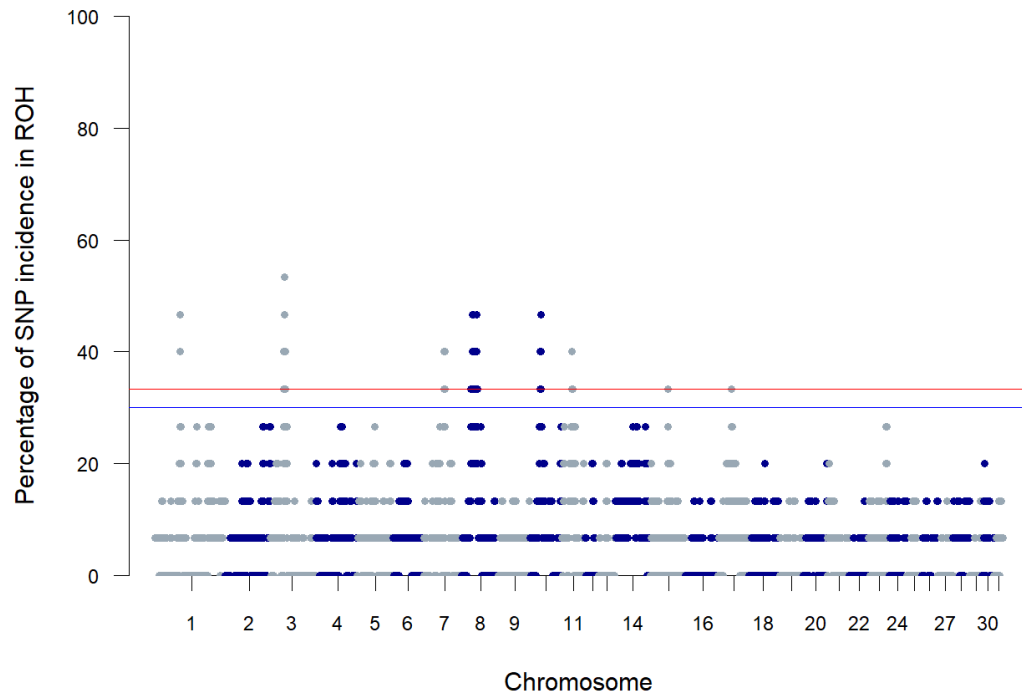

Supplement: Supplementary file 3 — Additional file 3. ROH islands of domestic horse breeds. Manhattan plots of incidence of SNPs appearing inside ROH for each of the 33 domestic horse breeds in the analysis. Abbreviated breed names and sample sizes are indicated in the top left corner of each plot. Horizontal lines indicate the breed-specific thresholds calculated based on standard normal z-scores generated from SNP-in-ROH incidence in 1 Mbp bins (red), and all SNP-in-ROH incidence (blue), above which ROH islands are indicated. In instances where only one line is visible, the values for the two thresholds are identical. [file 12864_2022_8729_MOESM3_ESM.pdf]

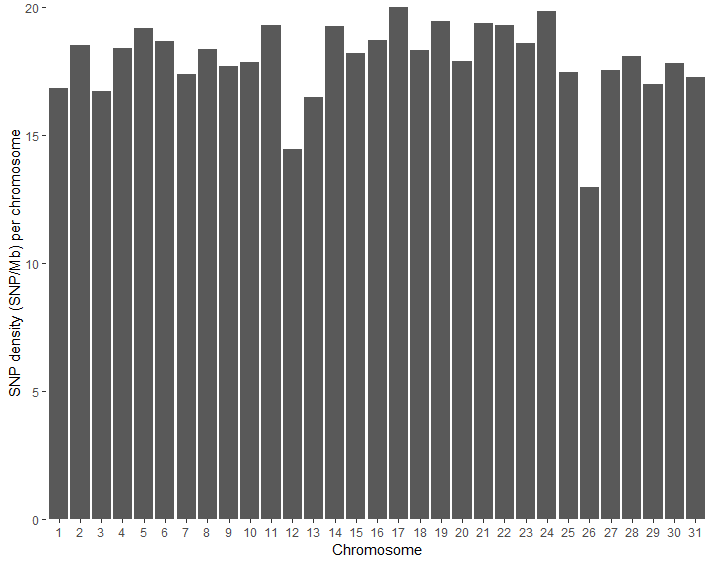

Supplement: Supplementary file 4 — Additional file 4. SNP density (SNP/Mb) per chromosome. Density of SNPs on each chromosome as calculated by the total number of SNPs used in the final dataset per chromosome divided by chromosome length in Mb. [file 12864_2022_8729_MOESM4_ESM.tiff]
